# Spatial distribution of health risk assessment of a drinking water reservoir exposed to urban agglomeration and industrial lead contamination in Istanbul, Türkiye

**DOI:** 10.1002/wer.70013

**Published:** 2025-02-03

**Authors:** Hüseyin Cüce, Erkan Kalipci, Fikret Ustaoğlu, Mehmet Ali Dereli, Seda Alkaya, Aysun Türkmen

**Affiliations:** ^1^ Department of Environmental Engineering Giresun University Giresun Türkiye; ^2^ Department of Geomatic Engineering Giresun University Giresun Türkiye; ^3^ Department of Biology Giresun University Giresun Türkiye; ^4^ Department of Environmental Engineering Nevşehir Hacı Bektaş Veli University Nevsehir Türkiye; ^5^ Department of Chemistry Giresun University Giresun Türkiye

**Keywords:** environmental risk, geographical analysis, harmful chemical inputs, Ömerli Dam, quality index

## Abstract

The Ömerli Dam, supplied by multiple rivers that provide a major amount of Istanbul's water demands for drinking and irrigation, is experiencing toxicological threats due to rapid expansion, increased industrial activity, and population growth in light of the impending global drought. In this study, multivariate statistical analysis and spatial distribution based on geographic information system (GIS) was used to examine the status of pollution and health risk, irrigation water quality, and stochastic geo‐environmental risk sources of 16 potentially toxic elements (PTEs: Na, Mg, K, Ca, Al, Cr, Mn, Fe, Co, Ni, Cu, Zn, As, Cd, Hg, and Pb) concentrations from samples in the dam. Also, spatial analyzing of quality indices was used to assess the dam's feasibility for irrigation and drinking. As per the results of the study, the dam water has mild heavy metal (loid) pollution, which is measured at 16 PTE by the heavy metal (loid) pollution index (HPI) and heavy metal (loid) evaluation index (HEI) values. The pollution level is low during the rainy season and moderate during the dry season. The water at sampling station S3 (HPI = 146.50) is unfit for consumption and cannot be used for drinking. The majority of harmful metal species exhibited elevated concentrations (notably Pb: 51.78 μg/L, Cr: 8.55 μg/L) in the dam lake water, particularly during the dry season. The toxic metals Pb and Cd account for 15.95% of the total variance, with a high degree of loading. Lead concentrations were found to be above WHO and USEPA limit values only in S2 and S4 during the wet season, but above 0.01 mg/L in all stations except S1 during the dry. Dam water is acceptable for agricultural use, according on the results of the, sodium absorption rate, the percentage of Na and the magnesium damage index, average SAR < 0.9, Na% < 25, and MH < 50, respectively. HQ_ingestion_ and HI values (all less than 1 in all PTEs) indicate that noncarcinogenic impacts might occur.

**Practitioner Points:**

- Ömerli Dam provides most of the drinking and potable water needs of a mega city like Istanbul.
- With this study, toxic metal pollution of dam surface water and its possible effects on human health were analyzed for the first time on a spatially wide scale.

## Graphical abstract

- The contents of 12 PTEs in the surface water of the dam lake were compared.
- Ecological risk indices and GIS‐based maps were used to assess stochastic health risks of PTEs.
- Factor/cluster and correlation analyses were conducted for source identification of PTEs.
- Lead levels in the dam surface water exceed the quality limits set by international organizations.

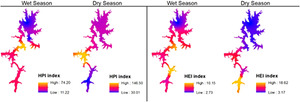

## INTRODUCTION

In recent years, there has been a notable increase in the contamination of natural environments with metals and metalloids on a global scale (Ma et al., 2021). Heavy metal (loid)s from areas exposed to anthropogenic chemical contamination pass from soil to surface waters through infiltration processes under the influence of climate change. Toxic accumulation may occur in water reservoirs due to chemical pollution caused by excessive amounts of various potentially toxic elements (PTEs)/heavy metal (loid)s seen in treated municipal wastewater, fertilizers, or polluted water. With this toxic waste carried by floods occurring near industrial areas or garbage storage areas, as well as agricultural areas, can negatively affect all living organisms. In terms of the environment, the metal fraction represents the bioavailable quote of the potentially harmful elements that can come into contact with the environment (soil or groundwater) and with food chains, thus defining the actual state of the pollution. The need to calculate post‐rainfall surface runoff, develop effective irrigation systems, manage water resources effectively, and safeguard the subsurface environment sustainably is growing in order to stop all possible causes of contamination (soil and water) in the environment (Al‐Bayati et al., 2024; Angelaki et al., 2022; Barbieri et al., 2020; Berkowitz et al., 2014; Selker & Assouline, 2017; Varol et al., 2022). Freshwater is becoming more and more necessary for the production of food and feed, as well as a variety of other goods, industrial activities, and meeting the demands of both urban and rural people. Freshwater ecosystems, including lakes, rivers, streams, and reservoirs, are among the most developed on Earth. However, they have been subjected to a variety of physical, chemical, and biological changes as a result of alterations in water flow, changes in land use and land cover, overharvesting, and increased harmful chemical inputs through sewage and fertilizers (Markad et al., 2021). A steady supply of water for residential, commercial, and agricultural use is ensured by reservoirs, which also help to alleviate the effects of water scarcity. These sources are impacted by various pollutants brought by runoff, and they are particularly affected if there is agricultural land adjacent to a water supply and an industrial zone adjacent to residential areas (Findik & Aras, 2023; Hoekstra et al., 2012; Okoye et al., 2021; Temizer et al., 2018) Unfortunately, in underdeveloped and emerging nations, 95% of home wastewater and 70% of industrial wastewater are released into the environment without being treated (Boateng et al., 2016; Varol et al., 2021). Because excessive sewage discharges from industries, farms, or households pollute utilized water sources when they become unacceptable.

One of the biggest problems impacting water quality is heavy metal (loid) pollution, according to experts (Withanachchi et al., 2018). In aquatic ecosystems, metals are typically contributed by a multitude of anthropogenic sources, including air deposition, overexploitation, mining, urban, agricultural, and industrial activities, as well as lithogenic sources, such as precipitation inputs, soil degradation, and rock disintegration. PTEs in water resources of both anthropogenic and natural origins lead to a decline in water quality. Those with a natural origin are typically caused by processes like bedrock erosion and volcanic eruption that occur within the basin's lithological and geological structure (Karadeniz et al., 2024; Köse et al., 2015; Yüksel et al., 2024). Due to their highly carcinogenic and poisonous, PTEs, which are among the more than 700 chemical contaminants identified in water bodies, are the most hazardous to the environment and people (Erdoğan et al., 2024; Proshad et al., 2019; Saleem et al., 2019; Ustaoğlu & Aydın, 2020). Among the essential elements for the healthy growth and development of the human body are iron (Fe), zinc (Zn), and manganese (Mn). However, excessive levels of these elements in water and food are toxic for human beings (Din et al., 2023; Muhammad et al., 2024). The discharge of PTE into aquatic systems has been demonstrated to exert a detrimental impact on lake ecosystems, disrupting the food chain and raising concerns about potential health risks. The existence of PTEs in aquatic environments that occur naturally is a serious problem since they can poison the variability of aquatic animals and habitats (Türkmen et al., 2005; Türkmen et al., 2009).

Lakes, and reservoirs represent a significant source of water for drinking and domestic purposes in remote rural and urban areas of developing countries (Haq et al., 2023; Nivesh et al., 2023). Consequently, the water characteristics of these water sources have been subjected to regular testing for contamination levels and risk indices (Xie & Ren, 2022), which serve as a valuable instrument for elucidating the perspectives of stakeholders, including the general public and policymakers, in decisions pertaining to its management and conservation (Pace et al., 2022). The distribution of dissolved metals in the water of natural lakes and dam lakes has been the subject of numerous studies (Cüce et al., 2020; Kalipci et al., 2017; Karadavut et al., 2012; Kumari & Hansdah, 2023; Qin & Tao, 2022; Tokatlı, Onur, Dindar, Malafaia, et al., 2023). However, as man‐made lakes, dam reservoirs have gotten less attention than natural lakes. Dam reservoirs endure significant water level variations due to the manipulation of inflow/outflow, which sets them apart from natural lakes. This raises the hydrodynamic circumstances' variability, which has a significant impact on how metals are distributed in dam reservoirs. Therefore, knowledge of the distribution of metals in dam reservoirs is required to enable efficient and long‐term water quality management.

It is imperative that measures be taken to address point and nonpoint source pollution in order to maintain the quality and potability of water resources, particularly in large metropolitan areas such as Istanbul. Istanbul, which is home to approximately 16 million people, is experiencing rapid population growth and industrialization. As a result, it is one of the fastest growing cities in the world, particularly in Europe. Indeed, the city's population growth rate is almost 18%, which is significantly higher than the national average (Alparslan et al., 2007; Güzel et al., 2022). In this study, the surface water quality and comprehensive environmental risk situation of Ömerli Reservoir one of the biggest dams in Istanbul's water supply were examined. The Ömerli Dam Lake area is one of the most polluted basins of Istanbul, with a population distribution that has increased by 4.6% annually over the course of the basin's history. The environmental assessment of the press emphasizes the important role of point sources, especially those of indigenous origin, as the most important sources of pollution. It is noteworthy that the population growth rate in the Ömerli Dam Lake basin is high, which has contributed to a decrease in water quality in the lake (Coskun & Alparslan, 2009). The goal was to find out how to preserve the basin's water resources in an environmentally responsible manner. This seasonal research focused on the Ömerli Dam Lake, the largest reservoir in the Marmara Region and the source of drinking water for Istanbul, the most developed and populated city in Türkiye with a population of almost 16 million. With a strategic location in the forested Ömerli Basin, the dam contributes significantly to the city's water management strategy. However, Ömerli Basin's natural habitats are seriously threatened by the fast settlement that occurred there after the 1990s, particularly in conjunction with the growth in population. The reservoir is the largest and most important reservoir among the many dams that supply water to Istanbul (such as Darlık, Terkos, Büyük Çekmece, Sazlıdere, and Kazandere Dam) (Albayrak, 2012; IEUPD, I. E. a. U. P. D., 2020). The Ömerli Dam reservoir not only contributes to Istanbul's water supply but also plays a key role in flood control and irrigation. Considerable nutrient dynamics research has been done in the region as a first step toward controlling lake pollution to identify the effect of waste loads on water quality of the dam lake. Although the consequences of heavy metal (loid) contamination in the reservoir and its surroundings have been studied, a thorough geographical/ecotoxicological health risk study has not yet been done. In addition to the effects of water scarcity brought on by global warming, the dam lake is also threatened by agrochemical pollution. Unfortunately, due to the limited number of multi‐dimensional and long‐term monitoring researches on the water quality of the dam, this study focuses on recognizing the extent to which the surface water of the reservoir has been impacted, particularly with regard to hazardous heavy metal (loid)s. The Ömerli Dam clearly shows the detrimental consequences of weather patterns, droughts, and other environmental activities that are present in many dam lakes around the world. Accordingly, this study represents the first in‐depth examination of harmful metal pollution in dam waters and its consequences on physical health.

The contamination of water sources with potentially harmful elements and other water quality parameters is the result of a combination of natural and anthropogenic factors. The erosion of mineral regions or ore deposits, a natural phenomenon known as lithogenic processes, has contributed to the deterioration of water quality. Additionally, the application of agricultural chemicals, the disposal of mining and industrial waste, and other activities associated with human development have also played a role in the contamination of water sources (Muhammad, 2023; Tokatlı, Varol, & Ustaoğlu, 2023). Water quality in dams should be evaluated frequently, the causes of pollution change should be determined, and appropriate actions should be taken to protect the ecological balance and ensure the sustainable use of water resources. In the assessment of the pollution of irrigation and drinking water sources, additional multivariate statistical methods that are commonly employed are ecological risk indices, pollution indexes, Pearson correlation matrix, principal component/factor analysis, and hierarchical cluster analysis (CA) (Akbal et al., 2011; Kalipci et al., 2023; Mohan et al., 1996; Tokatlı, Varol, Ustaoğlu, & Muhammad, 2023; Yazman et al., 2024). This study suggests an integrated spatial/statistical procedure to identifying the geochemical/toxicological assessment of metal contamination in the surface water of the reservoir. The assessment of pollution indices in conjunction with the suggested integrated method, allows to establish the surface water metal contamination status. Accordingly, this study represents a critical investigation into the harmful effects of metal contamination in the waters of the Ömerli Dam on human health. Over two seasons, field and laboratory studies were conducted to measure the concentration of toxic metals at six sampling points in the reservoir and to determine the extent to which anthropogenic activity or natural processes contributed to any enrichment that was noticed. The following objectives guided the year‐long research at the reservoir:

- Within the scope of the study, Pearson correlation matrix (PCM), principal component analysis (PCA), and cluster analysis (CA) were used as examples of multivariate statistical methods to identify possible sources of harmful elements.
- To ascertain the 12 PTEs' concentrations (Mn, Al, As, Cr, Zn, Fe, Ni, Cd, Co, Cu, Hg, and Pb), furthermore, a risk map of the entire dam was created by interpolating index values of ecotoxicological contamination and PTE distribution maps using a geographic information system (GIS).
- Utilizing indices like the heavy metal (loid) pollution index (HPI), heavy metal (loid) evaluation index (HEI), and water quality index (WQI) ascertain the ecotoxicological hazards posed by harmful elements.
- To assess irrigation water's suitability for use in agriculture based on factors like SAR, Na%, MH.
- To make a comparative evaluation by examining numerous studies published on the health risks of heavy metal (loid)s in dam water. The possible risks to children's and adults' health from exposure to hazardous metals were assessed using the Environmental Protection Agency (USEPA, 2018) risk model.

## MATERIAL AND METHODS

### Research area

Ömerli Reservoir, which has a maximum depth of 62 m and is located in the northeast of Istanbul (about 30 km away), was built on Riva River, the largest stream in Istanbul, which is roughly 100 km long. Data from the Government Water Works Department (DSI, Directorate General for State Hydraulic Works) show that the dam today provides 43% of Istanbul's drinking water demands and spans a basin area of 621 km^2^. The dam's construction was completed in 1987, and, since then, it has been a vital asset in managing the water resources of the region. The dam helps regulate the flow of the Göksu River, preventing potential flooding during periods of heavy rainfall. Additionally, the stored water is utilized for agricultural purposes, supporting local farming communities and contributing to the sustainability of the region's food production (Güler, 2010; Türker, 2002; UNDP, 2018). The quick growth of tiny communities, which leads to population growth and increased industrial activity, is the cause of the pollution issues at Ömerli Reservoir. Additionally, there are numerous smaller streams, which are discharging into the reservoir (Morkoç et al., 2009).

The dam, which has a maximum water storage capacity of 236 million m^3^ and a surface area of 31 km^2^, is the biggest reservoir for storing water sent to Istanbul from various sources. Istanbul is a mega city that can be under the influence of extreme drought especially in summer due to the effect of climate change and is trying to meet the needs of its limited water resources and overpopulation. The dam's role in supporting the city's water needs is particularly crucial during periods of drought or water scarcity. With climate change affecting weather patterns, Ömerli Dam becomes a key asset in Istanbul's resilience against the challenges posed by a changing climate. For this reason, it mostly depends on high‐capacity reservoirs for most of its urban, industrial, and irrigation needs. Apart from meeting the city's water requirements, the dam is essential for managing floods and assisting regional farming. Efficient and sustainable dam management is ensured by ongoing research and monitoring initiatives.

Ömerli Dam's critical role in supplying a dependable and safe water supply is becoming more and more apparent as Istanbul expands. The reservoir sustains the local ecology and provides a home for a variety of aquatic creatures. Furthermore, the Ömerli Basin is a sizable natural area with a wealth of biological variety, home to around 37 rare taxa. One of Türkiye's richest populations of several of these species is periodically included in the scope under the heading “Important Plant Areas.” The Polonezköy Nature Park and the Ömerli catchment provide some protection for the lands, which also comprise the largest Heathland areas in Eastern and Eastern Mediterranean Europe. However, the region is under severe threat as a result of the rapid growth of Istanbul's habitation areas, particularly in the southern regions. Agricultural land decreased by up to 82% between 1990 and 2010, while built areas increased 169%. Moreover, a highway passing through the region increased the pollution in the protected area. The short‐/medium‐/long‐distance protection zones contain industrial sectors even though the basin is a drinking water basin. Several industries, including those that process gypsum, concrete, glass, various metals, milk and dairy products, grains, textiles, detergents, synthetic products, and plastics, as well as inorganic chemicals used in the ingredients industry, are active throughout the basin. These industrial facilities are mostly involved in the metal sector (60 units), with poultry and fattening farms and their associated industrial establishments making up the second‐largest group (58 units). The third group is made up of facilities for the manufacturing of products from trees and forests (32 units) and facilities for the plastic and chemical industries (39 units) (Bayhan et al., 2017; Ceylan, 1999; Cüce, Kalipci, Ustaoğlu, Dereli, & Türkmen, 2022b; IEUPD, 2020; Temel, 2006; Tezer et al., 2015).

### Water sample collection and analytical techniques

**FIGURE 1:**
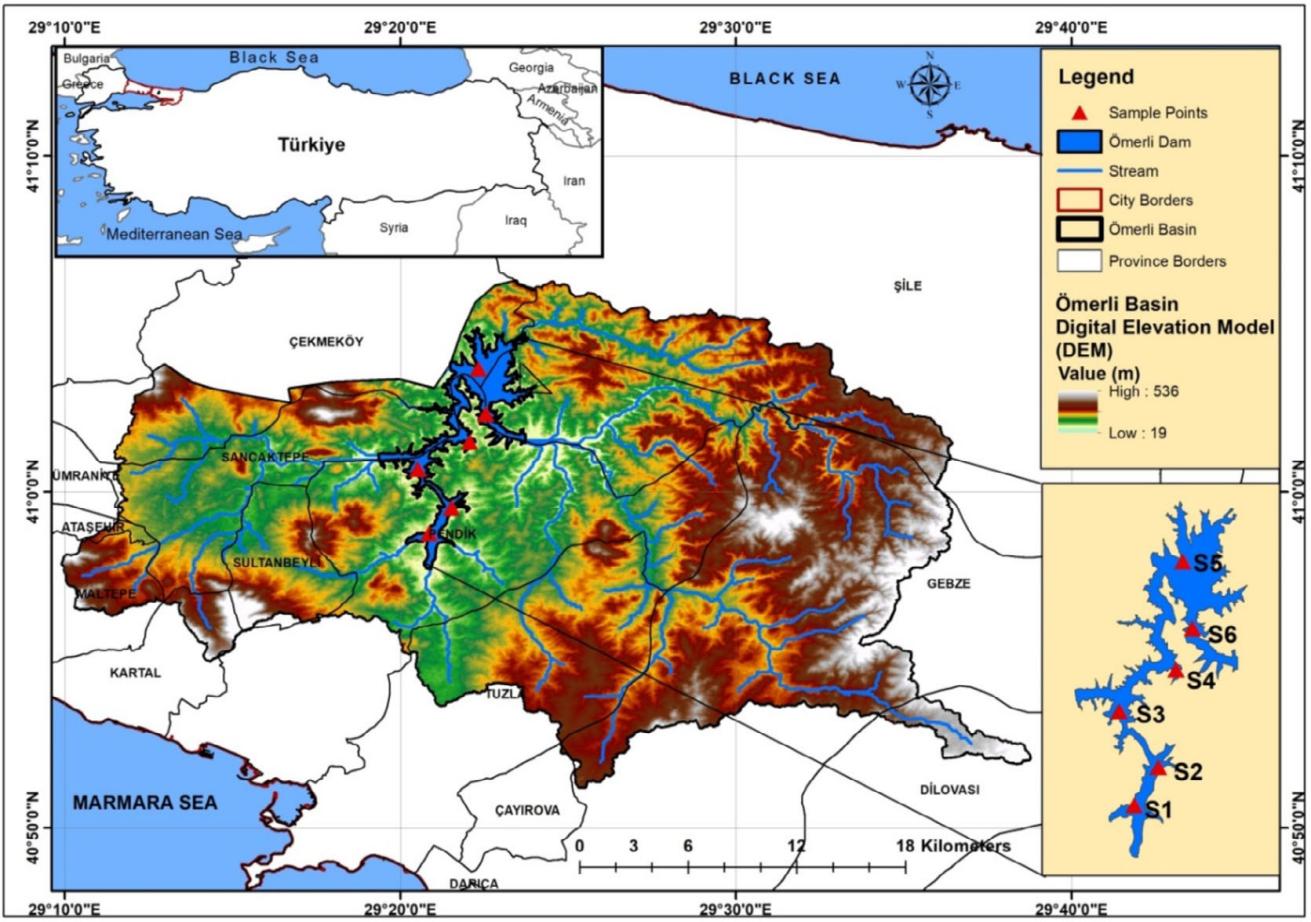
Sampling stations map of Ömerli Dam.

Two sets of dam water samples were obtained (in dry/wet seasons) from six sampling points in 2019–2020 (Figure 1). In accordance with the accepted methodology (APHA), composite surface water samples (comprising 2–5 subsample from at least two points) were collected in pre–cleaned PE bottles (1.5 L in capacity) at every sampling location where GPS (Magellan Explorist 710) was used to record the location. The sampling stations were chosen in order to offer exemplary data on PTEs concentrations in waters throughout Ömerli Dam such as pollution hotspots can be found in urban effluent areas, industrial/agricultural areas, and unregulated residential wastewater discharge canals.

Using Millipore nitrocellulose membrane filters with a 47 mm diameter and 0.45 μ pore size that had been acid‐washed, samples were instantly filtered in place. At each location, the following values were measured using the probe (Hach 40dQ model portable‐multiparameter): pH, water temperature (°C), DO (dissolved oxygen, mg/L), EC (conductivity, μS/cm), and salinity. Samples were brought into the lab and maintained at +4°C. Lab test kits (Hach‐Lange) were used to assess concentrations, BOD5, COD, SRP, TN, TSS, Cl‐, Chl_a, anionic surfactant, and TA (mg/L) in accordance with established methods (APHA, 2007). All analyses and spectrophotometric measurements were carried out using chemicals of analytical purity utilizing a thermo‐reactor (Hach‐Lange LT‐200), pH meter (Mettler Toledo Brand), analytical scales (BEL Brand accuracy), and Hach‐Lange DR3900.

All of water samples acidified with nitric acid (65%) placed into 10‐mL Teflon tubes in order to determine the quantities of soluble metals. An inductively coupled plasma mass spectrometer (ICP‐MS) manufactured by Agilent Technologies (Tokyo, Japan) was used to measure PTE concentrations. Standards for each metal were made from analytical pure solutions (with a precision and accuracy of roughly 95%), and Reference Material UME CRM1201 was used to validate all analytical data in order to calibrate the instrument.

### Geographical and statistical analytics

It is difficult to assess the degree of metal contamination in water supplies. Communities and the environment can be protected from harm by anticipating, preventing, and proactively managing threats by mapping vulnerability holistically and taking into account the intricate relationships between natural hazards. In order to study the toxicity of elements in the water column of reservoirs, a number of indices and maps have been presented. Researchers have utilized these indices, which are applied to lakes, river/river basins, and wetlands, in conjunction with other indices (Bakan et al., 2010; Brraich & Jangu, 2015; Kumwimba et al., 2016; Sahoo & Swain, 2020; Ustaoğlu et al., 2017) and a range of multi‐statistical techniques (Cüce, Kalipci, Ustaoğlu, Dereli, & Türkmen, 2022a; Cüce, Kalıpcı, Ustaoğlu, Kaynar, et al., 2022; Findik & Aras, 2023; Leventeli & Yalcin, 2021; Ustaoğlu et al., 2020) to assess the water quality and track changes in heavy metal (loid)s in the water source.

PCA was used to condense the data package and check for correlations between the suspected harmful components under this investigation. Additionally, Pearson correlation coefficients were computed in order to find any potential strong correlations between the PTEs under study. In research on environmental pollution, CA is a significant collection of multivariate statistical techniques that is frequently used to categorize the examined areas as parameter, temporal, and spatial (Alkarkhi et al., 2008; Aydin et al., 2021; Li, Gu, et al., 2008). The main goal of the multivariate technique (CA) is to organize the system's objects into groups or groupings according to commonalities. The objective is to identify the best possible clustering wherein each cluster's observations or items are similar but the clusters are different from one another. Most common statistical software tools (MINITAB 16.0) can easily be used to perform CA in this study. Additionally, for the creation of the index graphics, the statistical programs SPSS 22 and PAST 3X were employed.

ArcGIS 10.7 was used to conduct all spatial analysis. Using the distance‐weighted inverse distance weighting (IDW) interpolation approach, maps were created to evaluate the consequences of weather changes on the reservoir's water quality. Consideration was given to the IDW approach, a deterministic spatial interpolation method employed in the work (Lu & Wong, 2008). The points closest to the target location are given more weight using this strategy (Shukla et al., 2020). According to the opposite of the distance between points, each point's impact on the other is dependent (Tercan & Dereli, 2020). The sampling points that are nearest to the cell to be approximated are given a lot of weight. As a result, as the points move away from the expected position, their effects become less significant.

### Assessment of the dam water's quality

Ecotoxicological indices, such as WQI, HPI, and HEI, and assessments of health risks based on relative weight and toxicological parameters of the metals under investigation—listed in Table S1 and Table S2, were used to compute the water quality evaluation. Moreover, irrigation water quality of the Ömerli Dam was evaluated with the sodium absorption rate (SAR), %Na, and magnesium hazard (MH) parameters, which calculated. Provided in Supporting information are the equations utilized in the calculations. Standard operating methods, reagent blanks, and spiked sample recovery were used to assess the consistency of the data in order to ensure quality control and assurance.

## RESULTS AND DISCUSSION

### PTE concentrations and spatial analysis

Results of the investigation of physicochemical parameters, concentrations of four major elements (Na, Mg, K, and Ca) and 12 PTEs (Al, Cr, Mn, Fe, Co, Ni, Cu, Zn, As, Cd, Hg, and Pb) of the water samples collected from Ömerli Dam are summarized in Table S3. The PTEs' average concentrations belonging to the dam water samples in the wet‐dry periods are given in Table S4 compared to the World Health Organization (WHO, 2017), USEPA (2008), and TS266 (2005)‐established limit values. It was therefore concluded that, in comparison to the WHO values, the As concentration was relatively high. Nevertheless, Turkish surface water rules designated it as Class 2 water. It was found that Mn was marginally higher than the WHO‐recommended limit amount.

**FIGURE 2:**
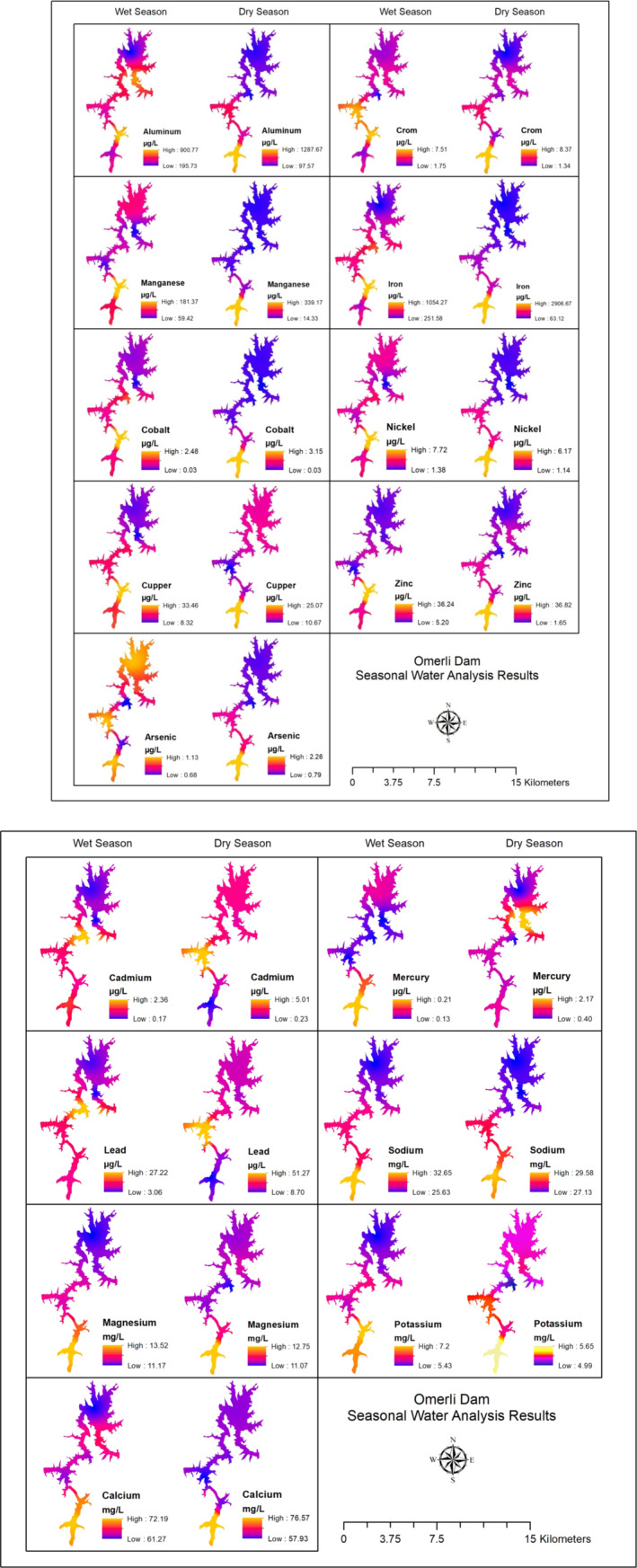
Seasonal geographical analysis of potentially toxic elements (PTEs) in the dam surface water sample points.

Potentially harmful elements were compared to those found in prior research in dam water samples collected from six sampling locations in the reservoir for this investigation (Table 1). Ömerli Dam area, like many other reservoirs throughout the Europe, is sincerely threatened by urbanization, agricultural pollution, and the majority of industry effluents. The different‐sized streams that are connected to the dam, such as a canal that, based on the inherent and man‐made impacts in their river basins, carries pollutants that discharge PTEs of various types and noticeably larger concentrations both directly and indirectly. These pollutants are carried from agricultural regions by rainwater washing them. Güzel et al. (2022) determined that the pollution in the Ömerli Dam Lake was petrogenic, as had been determined for other water resources in Istanbul (Terkos, Büyükçekmece, Elmalı, Darlık, Sazlıdere, Alibey) based on the molecular ratios they had established. The spatial analysis of 16 PTEs in the dam water samples is displayed in Figure 2 as seasonally. In this work, an uninterrupted surface corresponding to the researching area is created using the IDW technique, one of the interpolation methods. In the dam water body, the majority of the metals exhibited notable spatial fluctuations during the research periods. The southern part of the dam, where S1, S2, and S3 stations are located, has relatively higher accumulation of PTEs compared to the northern part (Figure 2). Hydrological impacts, lithological inputs, geological characteristics, cultural influences, and vegetation type changes can occasionally also have an impact on variations in metal concentrations in the Reservoir. Upon comparing Ömerli Dam's results with those of the other research projects (Table 1), it was observed that the mean As level (1.08 μg/L) was lower than all reservoirs except to Batman Dam, Türkiye. Also, compared to the Atatürk, Keban, Kralkızı, Dicle, and Batman dam reservoirs in Türkiye, the average concentrations of Cr, Ni, and Zn were found to be much lower in the Ömerli Dam reservoir. However, the detected Cd and Pb concentrations are higher than those measured at all other dams listed in Table 1 (except for Eğirdir Lake, Türkiye and Lake Texoma, USA). Furthermore, the mean Cu concentration (16.49 μg/L) is less than that observed in Atatürk and Keban dams. However, the opposite is true for the Fe and Mn elemental analysis results (Table 1). The anthropogenic sources of copper are represented by mining, the utilization of sewage sludge, waste emissions, and the intensive application of fungicides and fertilizers in agricultural regions. This is explained by the presence of settling processes and dilution, which move metals into the sediments. Also, increased precipitation in the winter reduced the overall metal concentrations in the Dam by allowing a significant amount of uncontaminated water to mix with contaminated water. However, due to the unexpectedly heavy summer rainfall, which resulted in huge water intakes, the reservoir's metal concentrations may have been recorded at low levels during the dry period, which had the lowest total metal concentrations.

**TABLE 1:** Comparison of determined potentially toxic element (PTE) levels (μg/L) with other monitoring studies.

	Al	Cr	Mn	Fe	Co	Ni	Cu	Zn	As	Cd	Pb	Hg
Ömerli Dam, Türkiye^a^	475.11	3.7	85.71	591.78	0.72	2.8	16.49	11.43	1.1	1.4	15.24	0.51
Atatürk Dam, Türkiye^b^	‐	‐	4.1	62	‐	15.4	25	64	‐	‐	‐	‐
Balaton Lake, Hungary^c^	‐	‐	‐	‐	0.14	9.2	5.1	126	‐	0.3	3.5	‐
Batman Dam, Türkiye^d^	‐	16.5	‐	57.7	‐	15.96	9.1	‐	0.7	0.1	‐	‐
Damsa Reservoir, Türkiye^e^	‐	6.3	6.5	‐	‐	2.1	270	2.5	8.3	<0.1	0.1	‐
Danjiangkou R., China^f^	‐	6.3	‐	19.2	‐	1.7	13.32	2.0	11.08	1.2	10.59	‐
Dicle Dam, Türkiye^g^	‐	18.58	‐	62.1	‐	15.86	2.1	‐	1.6	<0.13	‐	‐
Eğirdir Lake, Türkiye^h^	‐	5.1	70.8	771.8	‐	47.5	39.2	64.4	‐	2.2	15.3	‐
Ferneziu Lake, Romania^i^	11.9	0.3	415	511	0.3	1.6	0.7	1.8	0.2	0.1	0.8	‐
Keban Dam, Türkiye^j^	‐	79	‐	297	‐	3	408	565	‐	‐	‐	‐
Kışla Dam, Türkiye^k^	‐	‐	‐	4	‐	0.3	3.0	6.7	‐	0.14	0.6	<0.1
Kralkızı Dam, Türkiye^l^	‐	22.06	‐	58.6	‐	15.75	2.8	‐	2.4	<0.1	‐	‐
Lake Texoma, USA^m^	92	4	7	119	<2	5	24	59	<33	20	<15	‐
Lake Victoria, Kenya^n^	‐	‐	‐	‐	‐	‐	1.6	144	‐	<0.1	1.1	‐

^a^ Present study.

^b^ Karadede & Ünlü, 2000.

^c^ Nguyen et al., 2005.

^d^ Varol, 2013.

^e^ Findik & Aras, 2023.

^f^ Li, Xu, et al., 2008.

^g^ Varol, 2013.

^h^ Özçelik & Tekin‐Özan, 2024.

^i^ Dippong et al., 2024.

^j^ Canpolat, 2007.

^l^ Şimşek & Mutlu, 2023.

^l^ Varol, 2013.

^m^ An & Kampbell, 2003.

^n^ Outa et al., 2020.

### Ecotoxicological indexes of water quality

**FIGURE 3:**
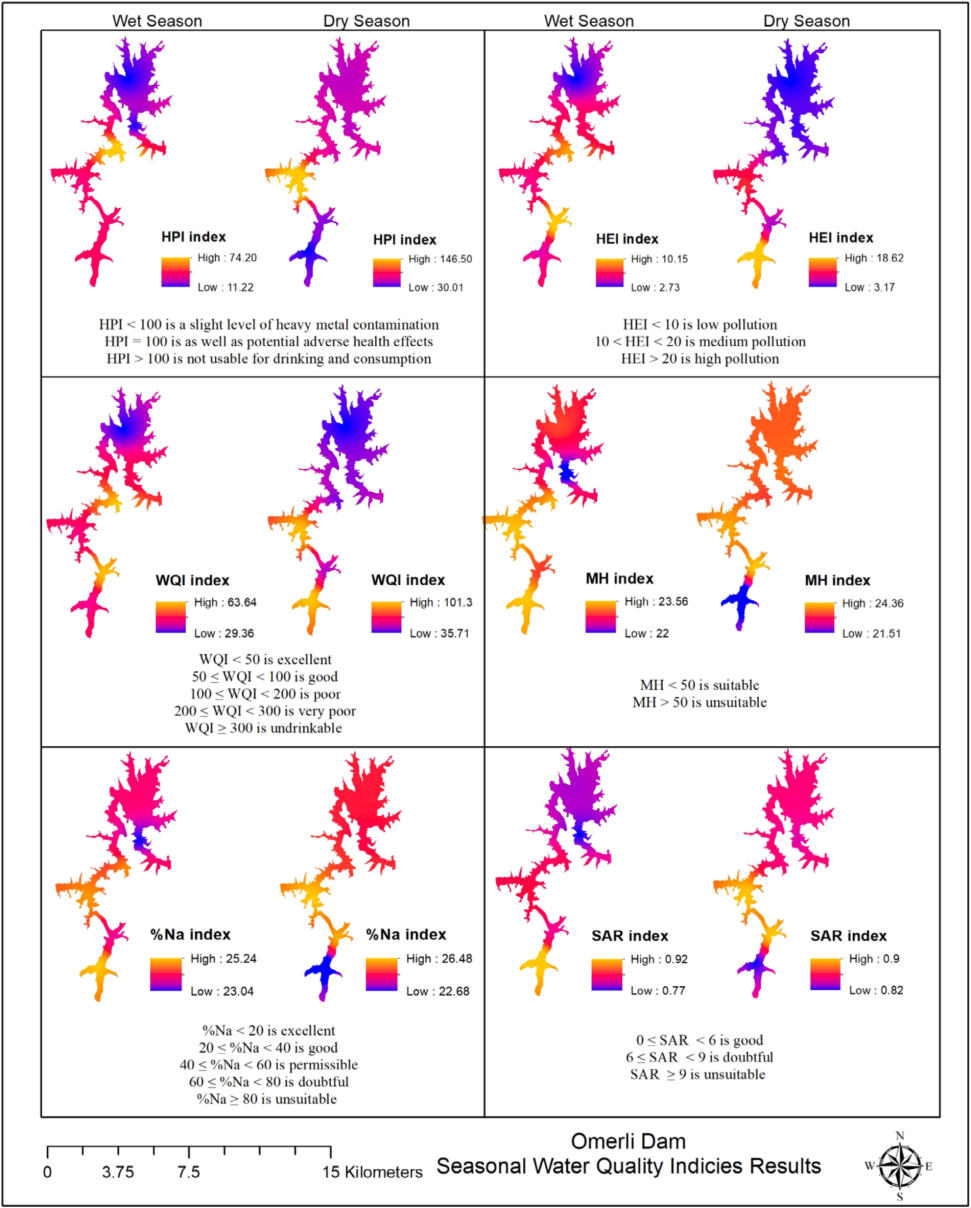
Seasonal geographical distribution of the dam surface water sample points by HEI, HPI, WQI, and irrigation water quality assessment by magnesium hazard (MH), %Na, and sodium absorption rate (SAR) indices.

A seasonally summarized geographical distribution of each parameter, derived from the HEI, HPI, and WQI ecotoxicological index values, is shown in Figure 3. Consequently, in this study, the WQI values calculated using Equation (1) in Supporting information ranged from 29.36 to 63.64 and 35.71 to 101.30 for surface water samples at wet and dry seasons, respectively. The wet period WQI average is 47.31, whereas the dry period average is 56.14. Based on the WQI findings, the water quality of the dam was found to be excellent in the rainy season and good in the dry season (Figure 3). However, it was determined that the quality of the dam surface water decreased from good to poor because the WOI values calculated at the S1 and S2 stations in the dry period were slightly above the limit value of 100 (101.30 and 100.32, respectively). There is a little amount of PTE contamination in the dam surface water due to average HPI <100 (31.28 and 60.46, respectively) during rainy and dry periods. The HPI value is just HPI >100 only at sampling station S3 (146.50) in the dry season. Consequently, it is not appropriate to drink the water at this sample site (S3) and should not be consumed. Since the mean HEIs <10 in the wet (6.16) and dry (7.59) seasons, the degree of dam water quality is evaluated as “low pollution” (Figure 3). However, because the HEI values calculated at stations S1 and S3 in the dry period were higher than 10 (18.62 and 11.51, respectively), the water quality was classified as “medium pollution.”

### Evaluation of irrigation water quality

Surface water outputs showed slightly alkaline characteristics with the mean pH value of 7.81 in wet season and 7.98 in dry season. Measurements made on‐site of the dam water's dissolved oxygen (DO) were recorded in the range of 7.68–10.55 mg/L. The mineral characteristic of the dam water was typically measured by TA and EC. TA values of the dam water were measured between 68 and 144 mg/L and EC 299–335 μS/cm. In the dry season, the average BOD value (7.09 mg/L) was more above the average WHO value (WHO, 2017) of 5 mg/L (Table S3).

Irrigation water chemistry varies with source and with regional differences in geology and climate. High salinity water, for instance, can be very detrimental to plants since it decreases their ability to absorb water, slows down their growth, and changes the way they use energy (Aydin et al., 2021). In the study, the indices of irrigation water quality, MH sodium percentage (Na%), and SAR, were seasonally calculated and shown in Figure 3 as spatial distribution. The results of the irrigation water quality were evaluated according to Table S5 (Ravikumar et al., 2013). Thus, the average MH parameter in the wet (23.12) and dry periods (23.45) was found to be ‘suitable’ in terms of irrigation water, because the mean MH parameter was less than 50. The quality of the water is in the “good range”, as the typical sodium content ranges from 20 to 40 in the wet (24.30) and dry (25.09) periods. Based on the mean salt absorption rate parameter during the wet (0.83) and dry periods (0.86), the dam water's quality was found to be in the “good” category. Significant changes in the physicochemical quality of water, such as persistent COD, high suspended solids, and excessive salinity, can be observed as a result of pollution from the arrangements surrounding the dam and channel connections, as well as the sensitivity of dam water to changes in water level. Because the waste load is greater than the receiving water environment's ability to clean itself, the resources feeding the dam lake and natural biological features have suffered. As a result, in recent years, incidents like mass fish fatalities have been caused by toxic effects and abrupt shifts in the trophic level of dam water.

### Risk evaluations for health

PTEs in water environments are taken up by creatures, which then spread infection to people via the food web and build up in tissues. As a result, a wide range of diseases, such as cancer, are possible and constitute a serious threat to people (Herojeet et al., 2015; Töre et al., 2021). Human exposure to potential toxic elements is an issue of great concern (Anyanwu et al., 2018). Ingestion and cutaneous pathways are the main ways that humans and other organisms are exposed to potentially hazardous elements in reservoir water. Because of this, the health risks—the Hazard Quotient (HQ), Hazard Index (HI), and Cancer Risk (CR) values—are computed and provided in Table 2. For the Dam water, HQ_ingestion_, HQ_dermal_, and HI values were all less than 1 in all PTEs in adults and children, suggesting that exposure to any negative health impacts is unlikely. During the dry and wet seasons, the total cancer risk (CR) for adults based on As (inorganic forms, which is classified as carcinogenic) and also other elements (Cd, Hg, and Pb) is less than the target risk level by international organizations. Therefore, there is no chance that adult exposure to dam water can cause cancer. However, these elements have no known benefits to the human body, but they are toxic even at low concentrations, and there is no known homeostasis mechanism for them (Forte & Bocca, 2011). The ingestion of waters with elevated concentrations of Cd can result in adverse effects on the livers and kidneys of fish and mammals. These effects may manifest as coronary heart disease, hypertension, and chronic lung disease in humans. At elevated concentrations, Pb can be toxic to the nervous system and kidneys and can also cause hypertension and damage the kidneys. Due to its oxidative degradation, high toxicity, and bioaccumulative characteristics, Pb has been linked to a range of irreversible health issues, including damage to the kidneys and reproductive organs, malformations, anemia, and cancer (Muneer et al., 2022).

**TABLE 2:** Hazard quotient (HQ), Hazard Index (HI), and cancer risk (CR) for elements in the Ömerli Dam in the dry and wet season.

WET			RfD_ing_	RfD_derm_		HQ_ing_		HQ_derm_		HI = ∑HQ_s_		Adult		
	(μg/L)	Kp	μg/kg/day	μg/kg/day	ABS_g_	Adult	Child	Adult	Child	Adult	Child	CR_ing_	CR_derm_	CR
Al	59.1	1.00E−03	1.00E+03	2.00E+02	2.00E−01	2.94E−03	3.29E−03	3.84E−04	8.49E−04	3.33E−03	4.14E−03			
Cr	4.86	1.00E−03	3.00E+00	7.50E−02	1.30E−02	5.08E−04	5.69E−04	8.16E−03	1.80E−02	8.66E−03	1.86E−02			
Mn	109.19	1.00E−03	2.40E+01	9.60E−01	6.00E−02	6.92E−03	7.75E−03	1.50E−02	3.33E−02	2.20E−02	4.10E−02			
Fe	38.22	1.00E−03	7.00E+02	1.40E+02	1.40E−02	2.96E−04	3.32E−04	5.53E−04	1.22E−03	8.49E−04	1.56E−03			
Co	0.07	4.00E−04	3.00E−01	6.00E−02	1.00E+00	7.95E−02	8.91E−02	8.30E−04	1.84E−03	8.04E−02	9.09E−02			
Ni	16.42	2.00E−04	2.00E+01	8.00E−01	4.00E−02	1.86E−04	2.08E−04	1.21E−04	2.68E−04	3.07E−04	4.76E−04			
Cu	0.65	1.00E−03	4.00E+01	8.00E+00	5.70E−01	7.39E−03	8.28E−03	3.38E−04	7.49E−04	7.73E−03	9.03E−03			
Zn	17.81	6.00E−04	3.00E+02	6.00E+01	2.00E−01	2.33E−04	2.61E−04	1.82E−05	4.03E−05	2.51E−04	3.01E−04			
As	28.61	1.00E−03	3.00E−01	2.85E−01	9.50E−01	8.79E−02	9.85E−02	5.08E−04	1.13E−03	8.84E−02	9.96E−02	3.96E−05	5.30E−07	4.01E−05
Cd	0.52	1.00E−03	5.00E−01	2.50E−02	5.00E−02	2.57E−03	2.88E−03	5.37E−03	1.19E−02	7.95E−03	1.48E−02			
Hg	0.42	1.00E−03	3.00E−01	2.10E−02	7.00E−02	1.03E−03	1.15E−03	1.10E−03	2.43E−03	2.13E−03	3.58E−03			
Pb	0.43	1.00E−04	1.40E+00	4.20E−01	1.17E−01	2.47E−02	2.77E−02	3.68E−04	8.14E−04	2.51E−02	2.85E−02			
DRY														
Al	58.62	1.00E−03	1.00E+03	2.00E+02	2.00E−01	2.49E−03	2.79E−03	3.25E−04	7.19E−04	2.81E−03	3.51E−03			
Cr	12.56	1.00E−03	3.00E+00	7.50E−02	1.30E−02	4.12E−04	4.61E−04	6.61E−03	1.46E−02	7.02E−03	1.51E−02			
Mn	60.76	1.00E−03	2.40E+01	9.60E−01	6.00E−02	5.33E−03	5.97E−03	1.16E−02	2.56E−02	1.69E−02	3.16E−02			
Fe	28.17	1.00E−03	7.00E+02	1.40E+02	1.40E−02	3.80E−04	4.25E−04	7.08E−04	1.57E−03	1.09E−03	1.99E−03			
Co	0.07	4.00E−04	3.00E−01	6.00E−02	1.00E+00	5.77E−02	6.46E−02	6.02E−04	1.33E−03	5.83E−02	6.59E−02			
Ni	14.12	2.00E−04	2.00E+01	8.00E−01	4.00E−02	1.38E−04	1.54E−04	8.99E−05	1.99E−04	2.28E−04	3.53E−04			
Cu	0.83	1.00E−03	4.00E+01	8.00E+00	5.70E−01	6.03E−03	6.76E−03	2.76E−04	6.11E−04	6.31E−03	7.37E−03			
Zn	7.21	6.00E−04	3.00E+02	6.00E+01	2.00E−01	2.03E−04	2.27E−04	1.59E−05	3.51E−05	2.18E−04	2.62E−04			
As	27.69	1.00E−03	3.00E−01	2.85E−01	9.50E−01	1.07E−01	1.19E−01	6.17E−04	1.36E−03	1.07E−01	1.21E−01	4.80E−05	6.43E−07	4.86E−05
Cd	1.93	1.00E−03	5.00E−01	2.50E−02	5.00E−02	5.28E−03	5.91E−03	1.10E−02	2.44E−02	1.63E−02	3.03E−02			
Hg	0.14	1.00E−03	3.00E−01	2.10E−02	7.00E−02	5.71E−03	6.39E−03	6.08E−03	1.35E−02	1.18E−02	1.98E−02			
Pb	1.19	1.00E−04	1.40E+00	4.20E−01	1.17E−01	4.80E−**02**	5.38E−02	7.14E−04	1.58E−03	4.88E−02	5.54E−02			

*Note*: *Risky values for HQ and HI are given in bold (>1); **Risky values for CR are given in bold (>0.0001).

During the study period, a significant amount of regional and seasonal variability was seen in most metals. It is thought that the low level of toxic metal pollution levels observed in the area, especially during the dry period, is due to the lithological composition of the basin. Nonetheless, during this time period, certain metals (Pb and Cd) showed higher compositions, which could be related to their mixed origins (both human and natural contributions). Drinking water exposure for an extended period of time may increase your risk of developing conditions like neuropathy, diabetes, skin lesions, hypertension, and cancer (He & Charlet, 2013; Xiao et al., 2019; Yu et al., 2007).

### Multivariate analytics

**FIGURE 4:**
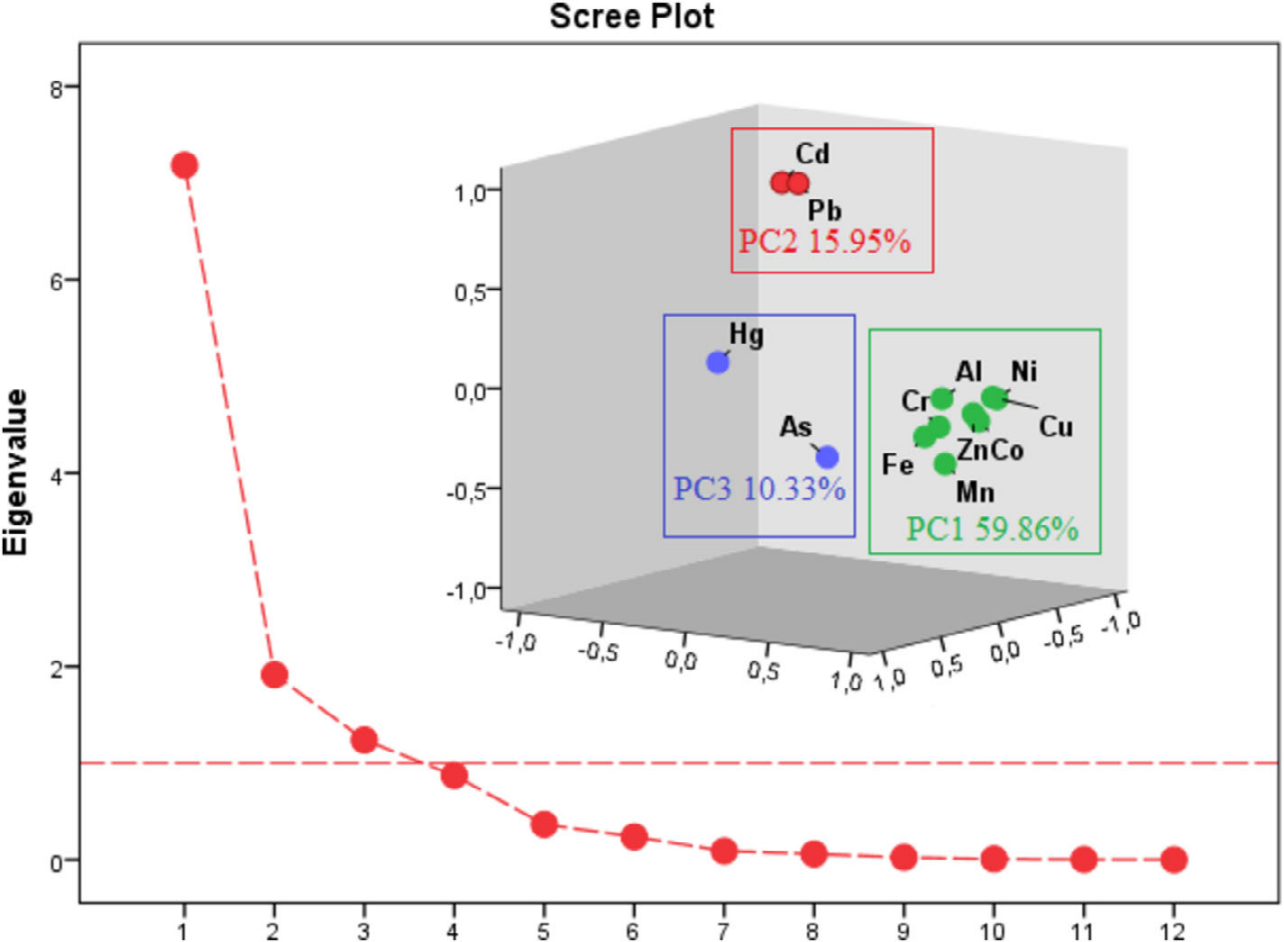
Principal component analysis (PCA) study of 12 potentially toxic elements (PTEs) in dam water samples.

**FIGURE 5:**
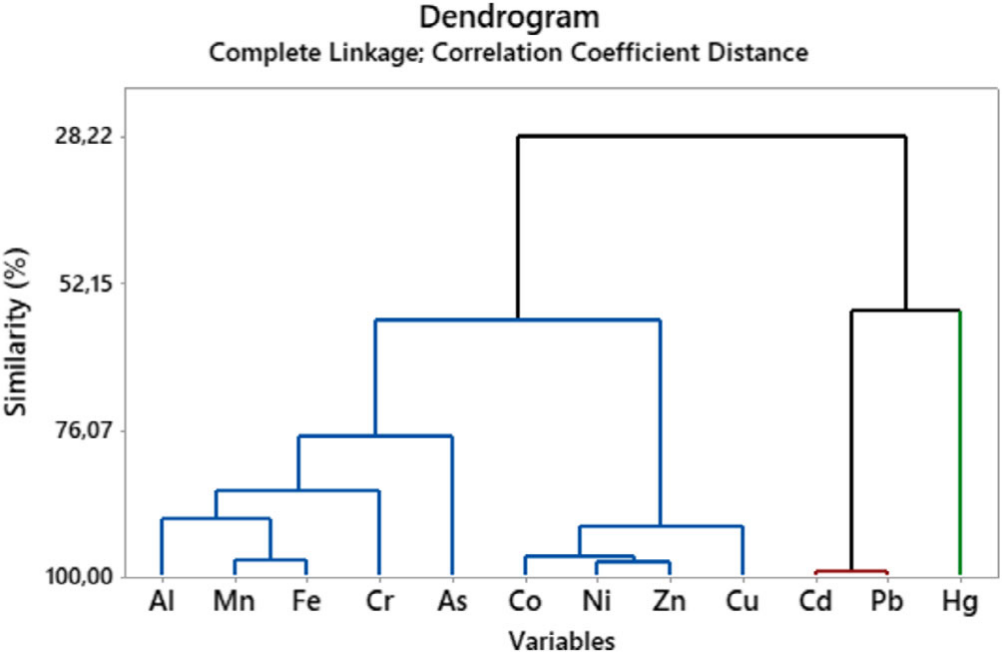
Dendogram for the clustering analysis of the 12 potentially toxic elements (PTEs).

**FIGURE 6:**
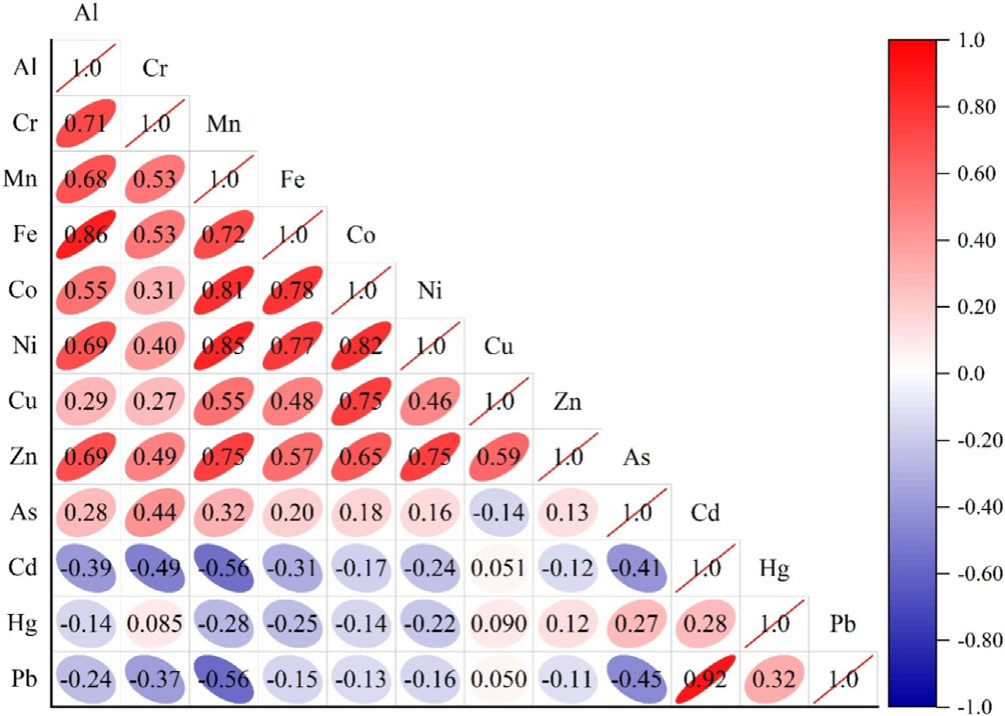
Matrix of Pearson correlation for 12 potentially toxic elements (PTEs) in the dam surface water points.

In environmental contamination research, CA, which is related to PCA, is frequently used to validate outcomes and facilitate variable categorization. With scores mostly contributing to environmental factors, principal component analysis (PCA) models were utilized to determine the sources of these PTEs in the dam water body through identify groups of variables (i.e., components) based on loadings. In order to maximize the variances between the variables under each component, PCA with varimax normalized rotation was used in this investigation. The variation diagram was shown in a rotated space in Figure 4, and Table 3 listed the PCA results using a varimax rotation technique for PTEs. Dendrograms, which show that areas within a cluster share similar traits and pollution sources, are frequently used as visual summaries of clustering techniques (Voza et al., 2015). In order to explore the parallels, the identical data were subjected to both PCA and CA in this study. Despite the applied methodology's seeming complexity, it frequently gives decision‐makers in statistical analysis and water quality management a tool with which to manage and assess a broad range of values. To evaluate better possibilities for managing the reservoir, these data need to be analyzed against worldwide standards. Potentially harmful materials were gathered inside the identical factorial group/cluster (Figure 4) as shown in the graph of dendrogram (Figure 5), which was produced by contaminating sources with similar features. Co, Ni, Zn, Cu, Al, Mn, Fe, Cr, and As make up the first cluster; Cd, Pb, and Hg make up the second; and As, Cd, and Ni make up the third. Bartlett's sphericity (BSP) test and Kaiser–Meyer–Olkin (KMO) test were performed before using PCA (Haghnazar et al., 2023). According to the results of the tests (BSP = 0 and KMO = 0.6), the concentrations of PTEs in the dam surface water sample points are appropriate for PCA. Outputs of the CA so supported the PCA findings. Additionally, PTEs belonging to the same component and cluster showed strong connections with one another (Figure 6). The outcomes showed that PCA whittled down the number of variables to three (PC1, PC2, and PC3), which explain 86.15% of the data variance. The PC1, strongly loaded by Co, Zn, Ni, Mn, and Fe and comparatively by Al, Cu, and Cr, accounted for 59.86% of the overall deviation. The PC2 accounted for 15.95% of the entire variance with heavy loading on Pb and Cd. The PC3 that correlated strongly with As and Hg accounted for 10.33% of the entire variance (Table 3).

**TABLE 3:** Potentially toxic elements (PTEs)' rotated component matrix from the dam's surface waters.

	PC1	PC2	PC3
Co	**0.971**	−0.117	−0.027
Zn	**0.966**	−0.074	0.013
Ni	**0.944**	−0.037	−0.224
Mn	**0.911**	−0.309	0.175
Fe	**0.897**	−0.151	0.332
Al	**0.881**	0.013	0.16
Cu	**0.825**	−0.06	−0.354
Cr	**0.817**	−0.144	0.096
Pb	−0.045	**0.989**	0.076
Cd	−0.174	**0.974**	0.033
As	0.557	−0.237	**0.686**
Hg	−0.112	0.169	**0.673**
Eigenvalues	7.18	1.91	1.24
% of variance	59.86	15.95	10.33
Cumulative %	59.86	75.81	86.15

Metal element correlation was also seen in samples of dam water. Figure 6 illustrates the substantial positive correlation (r = 0.92) between Cd and Pb, couple of the harmful heavy metal (loid)s under analysis. The concentration of Cd was below 1.5 μg/L. Cd is a naturally occurring element in the Earth's crust, occurring regularly as a mineral with other elements. Pb interferes with the assimilation and fixation of carbon, impeding the transfer of electrons in photosynthesis and damaging the reaction centers of phytoplankton cells (Wang et al., 2022). There are favorable interactions between Al, Mn, Ni, and Zn and many other metals. Because anthropogenic sources were included, it is likely that Cd, Hg, and Pb exhibited poor relationships with other PTEs (apart from copper).

The significance of the Ömerli Dam in the growth of Istanbul remains intact as the city keeps growing and developing. Istanbul needs to continue making regular improvements and maintenance work in the dam as well as infrastructural investments if it is to remain efficient in meeting water demands. The Reservoir serves as an example of effective water management policy for other regions facing comparable issues and is proof of the significance of sustainable water management in metropolitan settings. However, it increases the number of toxic metals (especially Cd and Pb) in the Ömerli Dam since it is a source of metal within the industrial complex, which encompasses over 60 metal industry facilities, as well as the Trans‐European Motorway (TEM). (Cüce, Kalipci, Ustaoğlu, Dereli, & Türkmen, 2022a). The Ömerli Dam clearly shows the detrimental consequences of weather patterns, droughts, and other environmental activities that are present in many dam lakes (Dippong et al., 2024; Markad et al., 2021; Özçelik & Tekin‐Özan, 2024) around the world. Thus, the findings have significant implications for scientists conducting research in this important field.

When the study's findings are analyzed, it is advised that fertilizers made of organic materials be widely applied, along with good agricultural practices, in order to protect both surface and ground water in all other dam basins in rapidly growing and increasingly populated cities that are at risk of water scarcity. Furthermore, it would be beneficial for local governments to educate the residents near the dam who use the dam water for irrigation and drinking about the negative effects of some PTEs mixing with surface waters.

The outcomes of the quality analysis in this research indicate that the rock–water interaction is responsible for the seasonal variations in the physicochemical status of the water samples. This is supported by the physical state and major ions found in the dam's water samples. Rainfall and seasonal human activity therefore have a significant impact on the surface water metal concentrations of this dam. Most of the metals revealed comparatively higher levels during the dry season. Lead metal, which is observed at a high‐risk level for Ömerli Dam, naturally originates from the bedrock ore but is rarely found in natural surface water resources and is detected in low concentrations. The main source of lead in dam water is the corrosion of plumbing materials containing lead components such as pipes, solder, taps, fittings, and old galvanized well coatings used in the infrastructure, depending on the rate of urbanization, and the mixing of these drainage waters into the reservoir through channels.

## CONCLUSION

Important conclusions were thus found for scientists working on various projects in this crucial area. Nearly, all harmful metal species had higher concentrations (especially Pb:51.78 μg/L, Cr:8.55 μg/L) in dam lake water, particularly during the dry season. This increase might be brought on by the drainage water returning from agricultural irrigation, as well as the wastewater discharged from the side branches that supply water to the dam and the locations where the channels connected to the dam meet. We identified Pb and Cr as possible contaminants of concern in the reservoir based on comparison with water quality requirements. The quality suitability for use/drinking of surface water in the reservoir was assessed using the ecotoxicological quality indices, HEI, HPI, and WQI. These techniques are helpful in reducing uncertainty in the choice of parameters, creation of rating curves, and determination of parameter weights. In both wet and dry conditions, all of the water samples extracted from the reservoir correspond to “good water,” based on the WQI values. This shows that both seasons had generally good water quality based on the sampled water. If the partially, bad water quality status at stations S1 and S3 is disregarded throughout the dry season. However, when the Pb, Fe, and Al metal analysis findings in the dam water samples are contrasted with the WHO's (2017) limit levels, it can be resulted that it is unfit to be consumed as water. The mean HPI readings for both the rainy and dry periods indicate the presence of slight contamination of PTE in the dam (annual mean 45.8). The quality of the dam water is classified as “low pollution” based on the HEI levels, with an annual mean of 6.8. The greatest WQI, HPI, and HEI values were observed at Station S4 during the rainy period, while Station S3 exhibited the highest values during the dry period. It can therefore be surmised that intense human‐induced activities, including industrial, agricultural, and domestic practices, as well as natural processes such as tourism, are responsible for the observed increase in PTEs. Furthermore, the sodium percentage (%Na), SAR value (annual mean 0.85), and MH index value (annual mean 23.3), which are used to assess the suitability of water samples for agricultural irrigation, demonstrate that the vast majority of these fluids satisfy the requirements for usage as irrigation water.

Because of the region's geological makeup and the chemicals used in agriculture, lead, cadmium, and mercury pollution in the study area is thought to be both anthropogenic and geogenic. A modest amount of carcinogenic risk is predicted to occur based on the adult cancer risk (CR) value resulting from this toxic element concentration. There is a possibility of noncarcinogenic effects, according to HQ_ingestion_ and HI values. Periodic monitoring of the water quality, identification of the causes influencing pollution changes, and implementation of appropriate mitigation strategies are necessary to preserve the ecological stabilize and protect water resources. In future studies, new and updated quality indices and statistical evaluations can be employed for the preservation of drinking and utility water resources, including dams that are similar to this one. Given the impending drought, it may be challenging to conduct additional study using geographic information system (GIS) and AI‐based analytics to identify environmental factors influencing hazardous pollutants in Ömerli Dam. To address the intricacy of the relationships between related factors and enable the linearization of particular water quality metrics, artificial intelligence and machine learning approaches can be applied.

## AUTHOR CONTRIBUTIONS

**Hüseyin Cüce:** Investigation; writing—original draft; methodology. **Erkan Kalipci:** Writing—review and editing; data curation. **Fikret Ustaoğlu:** Conceptualization; formal analysis; visualization; writing—review and editing. **Mehmet Ali Dereli:** Visualization; software; writing—review and editing. **Seda Alkaya:** Investigation. **Aysun Türkmen:** Validation.

## CONFLICT OF INTEREST STATEMENT

The authors declare that they have no known competing financial interests or personal relationships that could have appeared to influence the work reported in this paper.

## Supporting information


**Table S1.** Relative weight of each heavy metal.
**Table S2.** Toxicological parameters of the investigated metals used for health risk assessment according to USEPA (2008) (Wang et al., 2017).
**Table S3.** Minimum, maximum and mean concentrations of metal in the Ömerli Dam water.
**Table S4.** Permissible limits for surface waters according to standards of the Turkish Standard Institute surface water regulations, WHO (2017) and USEPA (2008).
**Table S5.** Quality classes according to irrigation water indices (Na%, SAR, and MH).

## Data Availability

Not applicable.

## References

[wer70013-bib-0001] Akbal, F. , Gürel, L. , Bahadır, T. , Güler, İ. , Bakan, G. , & Büyükgüngör, H. (2011). Multivariate statistical techniques for the assessment of surface water quality at the mid‐black sea coast of Turkey. Water, Air, & Soil Pollution, 216, 21–37. 10.1007/s11270-010-0511-0

[wer70013-bib-0002] Al‐Bayati, A. I. N. , Özkoç, H. S. , Burhan Mustafa, L. , & Özkoç, I. (2024). The effect of heavy metals on bacterial community structure and functional profile in surface soils of Baiji oil refinery and Baghdad City, Iraq. Geomicrobiology Journal, 41(1), 48–60. 10.1080/01490451.2023.2270482

[wer70013-bib-0003] Albayrak, İ. (2012). Applicability of the basin management model based on ecosystem services in the Istanbul‐Ömerli basin example', PhD (p. 224). Graduate School of Natural and Applied Sciences, Istanbul Technical University.

[wer70013-bib-0004] Alkarkhi, F. A. , Ismail, N. , & Easa, A. M. (2008). Assessment of arsenic and heavy metal contents in cockles (Anadara granosa) using multivariate statistical techniques. Journal of Hazardous Materials, 150, 783–789. 10.1016/j.jhazmat.2007.05.035 17590506

[wer70013-bib-0005] Alparslan, E. , Aydöner, C. , Tufekci, V. , & Tüfekci, H. (2007). Water quality assessment at Ömerli Dam using remote sensing techniques. Environmental Monitoring and Assessment, 135(1), 391–398. 10.1007/s10661-007-9658-6 17345006

[wer70013-bib-0006] An, Y.‐J. , & Kampbell, D. H. (2003). Total, dissolved, and bioavailable metals at Lake Texoma marinas. Environmental Pollution, 122, 253–259. 10.1016/S0269-7491(02)00291-9 12531314

[wer70013-bib-0007] Angelaki, A. , Dionysidis, A. , Sihag, P. , & Golia, E. E. (2022). Assessment of contamination management caused by copper and zinc cations leaching and their impact on the hydraulic properties of a Sandy and a loamy clay soil. Land, 11, 290. 10.3390/land11020290

[wer70013-bib-0008] Anyanwu, B. O. , Ezejiofor, A. N. , Igweze, Z. N. , & Orisakwe, O. E. (2018). Heavy metal mixture exposure and effects in developing nations: An update. Toxics, 6, 65. 10.3390/toxics6040065 30400192 PMC6316100

[wer70013-bib-0009] APHA, AWWA, WEF . (2007). Standard methods for the examination of water and wastewater'. American Public Health Association, American Water Works Association, and Water Environment Federation.

[wer70013-bib-0010] Aydin, H. , Ustaoğlu, F. , Tepe, Y. , & Soylu, E. N. (2021). Assessment of water quality of streams in Northeast Turkey by water quality index and multiple statistical methods. Environmental Forensics, 22, 270–287. 10.1080/15275922.2020.1836074

[wer70013-bib-0011] Bakan, G. , Özkoç, H. B. , Tülek, S. , & Cüce, H. (2010). Integrated environmental quality assessment of the Kızılırmak River and its coastal environment. Turkish Journal of Fisheries and Aquatic Sciences, 10, 453–462. 10.4194/trjfas.2010.0403

[wer70013-bib-0012] Barbieri, M. , Andrei, F. , Nigro, A. , Vitale, S. , & Sappa, G. (2020). The relationship between the concentration of rare earth elements in landfill soil and their distribution in the parent material: A case study from Cerreto, Roccasecca, Central Italy. Journal of Geochemical Exploration, 213, 106492. 10.1016/j.gexplo.2020.106492

[wer70013-bib-0013] Bayhan, H. , Erguven, G. O. , Akkoyunlu, A. , & Kanat, G. (2017). The assessment of water quality in Omerli dam reservoir, Istanbul, Turkey. Fresenius Environmental Bulletin, 26(1a), 977–988.

[wer70013-bib-0014] Berkowitz, B. , Dror, I. , & Yaron, B. (2014). Sorption, retention, and release of contaminants. In contaminant geochemistry: Transport and fate in the subsurface environment (2th ed.) (pp. 93–126). Springer. 10.1007/978-3-642-54777-5_2

[wer70013-bib-0015] Boateng, T. K. , Opoku, F. , Acquaah, S. O. , & Akoto, O. (2016). Groundwater quality assessment using statistical approach and water quality index in Ejisu‐Juaben municipality, Ghana. Environmental Earth Sciences, 75, 489. 10.1007/s12665-015-5105-0

[wer70013-bib-0016] Brraich, O. S. , & Jangu, S. (2015). Evaluation of water quality pollution indices for heavy metal contamination monitoring in the water of Harike wetland (Ramsar site), India. International Journal of Scientific and Research Publications, 5, 1–6.

[wer70013-bib-0017] Canpolat, Ö. (2007). The effects of polluting sources on water quality, sediment and reproduction biology and growth of Capoeta trutta (Heckel, 1843) in Keban Dam Lake (PhD thesis). Fırat Unıv. Graduate School of Natural and Applied Sciences, 336 pp.

[wer70013-bib-0018] Ceylan, M. (1999). Eutrophication in Ömerli Dam and its effect on the dam Lake and of affiliated streams, Master. Institute of Engineering and Science, Gebze Institute of Technology.

[wer70013-bib-0019] Coskun, H. G. , & Alparslan, E. (2009). Environmental modelling of Omerli catchment area in Istanbul, Turkey using remote sensing and GIS techniques. Environmental Monitoring and Assessment, 153, 323–332. 10.1007/s10661-008-0358-7 18537048

[wer70013-bib-0020] Cüce, H. , Kalipci, E. , Tas, B. , & Yılmaz, M. (2020). Evaluation of the impacts on water quality from meteorological changes due to differences in altitude by GIS: A comparison for two morphologically Different Lakes. The Black Sea Journal of Sciences, 10(1), 1–26.

[wer70013-bib-0021] Cüce, H. , Kalipci, E. , Ustaoğlu, F. , Dereli, M. A. , & Türkmen, A. (2022a). Integrated spatial distribution and multivariate statistical analysis for assessment of ecotoxicological and health risks of sediment metal contamination, Ömerli Dam (Istanbul, Turkey). Water, Air, & Soil Pollution, 233, 199. 10.1007/s11270-022-05670-1

[wer70013-bib-0022] Cüce, H. , Kalipci, E. , Ustaoğlu, F. , Dereli, M. A. , & Türkmen, M. (2022b). Multivariate statistical and spatial assessment of water quality from a dam threatened by drought at the mid‐Anatolia, Cappadocia/Turkey. Arabian Journal of Geosciences, 15, 441. 10.1007/s12517-022-09734-8

[wer70013-bib-0023] Cüce, H. , Kalıpcı, E. , Ustaoğlu, F. , Kaynar, İ. , Baser, V. , & Türkmen, M. (2022). Multivariate statistical methods and GIS based evaluation of the health risk potential and water quality due to arsenic pollution in the Kızılırmak River. International Journal of Sediment Research, 37, 754–765. 10.1016/j.ijsrc.2022.06.004

[wer70013-bib-0024] Din, I. U. , Muhammad, S. , Rehman, I. U. , & Tokatli, C. (2023). Spatial distribution of potentially toxic elements contaminations and risk indices of water and sediments in the Darband and Samana streams, Pakistan. Environmental Monitoring and Assessment, 195(11), 1343. 10.1007/s10661-023-11914-2 37858010

[wer70013-bib-0025] Dippong, T. , Senila, M. , Cadar, O. , & Resz, M. A. (2024). Assessment of the heavy metal pollution degree and potential health risk implications in lakes and fish from northern Romania. Journal of Environmental Chemical Engineering, 12(2), 112217. 10.1016/j.jece.2024.112217

[wer70013-bib-0026] Erdoğan, A. , Şeker, M. E. , Yüksel, B. , Ustaoğlu, F. , & Yazman, M. M. (2024). Elemental composition and nutritional values of chocolate bars available in Turkish markets: An integrated health risk assessment study. Journal of Food Composition and Analysis, 135, 106629. 10.1016/j.jfca.2024.106629

[wer70013-bib-0027] Findik, Ö. , & Aras, S. (2023). Application of the metal pollution indices on surface waters for assessment of environmental risk: A case study for Damsa reservoir (Cappadocia, Türkiye). International Journal of Environmental Science and Technology, 20, 1689–1698. 10.1007/s13762-022-04102-1

[wer70013-bib-0028] Forte, G. , & Bocca, B. (2011). Environmental contaminants: heavy metals. In Handbook of analysis of edible animal by‐products (pp. 403–440). CRC Press Boca Raton.

[wer70013-bib-0029] Güler, I. (2010). The impact of water use on agriculture: A case study from the Ömerli watershed. Water Resources Management, 24(10), 2101–2116.

[wer70013-bib-0030] Güzel, B. , Canlı, O. , & Aslan, E. (2022). Spatial distribution, source identification and ecological risk assessment of POPs and heavy metals in lake sediments of Istanbul, Turkey. Marine Pollution Bulletin, 175, 113172. 10.1016/j.marpolbul.2021.113172 34844748

[wer70013-bib-0031] Haghnazar, H. , Belmont, P. , Johannesson, K. H. , Aghayani, E. , & Mehraein, M. (2023). Human‐induced pollution and toxicity of river sediment by potentially toxic elements (PTEs) and accumulation in a paddy soil‐rice system: A comprehensive watershed‐scale assessment. Chemosphere, 311, 136842. 10.1016/j.chemosphere.2022.136842 36273611

[wer70013-bib-0032] Haq, A. U. , Muhammad, S. , & Tokatli, C. (2023). Spatial distribution of the contamination and risk assessment of potentially harmful elements in the Ghizer River basin, northern Pakistan. Journal of Water and Climate Change, 14(7), 2309–2322. 10.2166/wcc.2023.056

[wer70013-bib-0033] He, J. , & Charlet, L. (2013). A review of arsenic presence in China drinking water. Journal of Hydrology, 492, 79–88. 10.1016/j.jhydrol.2013.04.007

[wer70013-bib-0034] Herojeet, R. , Rishi, M. S. , & Kishore, N. (2015). Integrated approach of heavy metal pollution indices and complexity quantification using chemometric models in the Sirsa Basin, Nalagarh valley, Himachal Pradesh, India. Chinese Journal of Geochemistry, 34, 620–633. 10.1007/s11631-015-0075-1

[wer70013-bib-0035] Hoekstra, A. Y. , Mekonnen, M. M. , Chapagain, A. K. , Mathews, R. E. , & Richter, B. D. (2012). Global monthly water scarcity: Blue water footprints versus blue water availability. PLoS ONE, 7, e32688. 10.1371/journal.pone.0032688 22393438 PMC3290560

[wer70013-bib-0036] IEUPD, I. E. a. U. P. D . (2020). Istanbul Province 2019 environmental status report (p. 316). Ministry of Environment, Urbanisation and Climate Change.

[wer70013-bib-0037] Kalipci, E. , Cüce, H. , & Toprak, S. (2017). Spatial analysis of Damsa Dam Nevşehir surface water quality with geographic information system. Karaelmas Science and Engineering Journal, 7, 312–319.

[wer70013-bib-0038] Kalipci, E. , Cüce, H. , Ustaoğlu, F. , Dereli, M. A. , & Türkmen, M. (2023). Toxicological health risk analysis of hazardous trace elements accumulation in the edible fish species of the Black Sea in Türkiye using multivariate statistical and spatial assessment. Environmental Toxicology and Pharmacology, 97, 104028. 10.1016/j.etap.2022.104028 36455837

[wer70013-bib-0039] Karadavut, S. , Delibas, L. , Kalipci, E. , Ozdemir, C. , & Karadavut, I. S. (2012). Evaluation of irrigation water quality of Aksaray region by using geographic information system. Carpathian Journal of Earth and Environmental Sciences, 7, 171–182.

[wer70013-bib-0040] Karadede, H. , & Ünlü, E. (2000). Concentrations of some heavy metals in water, sediment and fish species from the Atatürk dam Lake (Euphrates), Turkey. Chemosphere, 41, 1371–1376. 10.1016/S0045-6535(99)00563-9 11057573

[wer70013-bib-0041] Karadeniz, S. , Ustaoğlu, F. , Aydın, H. , & Yüksel, B. (2024). Toxicological risk assessment using spring water quality indices in plateaus of Giresun Province/Türkiye: A holistic hydrogeochemical data analysis. Environmental Geochemistry and Health, 46, 285. 10.1007/s10653-024-02054-8 38967745 PMC11226512

[wer70013-bib-0042] Köse, E. , Çiçek, A. , Uysal, K. , Tokatlı, C. , Emiroğlu, Ö. , & Arslan, N. (2015). Heavy metal accumulations in water, sediment, and some cyprinid species in Porsuk stream (Turkey). Water Environment Research, 87, 195–204. 10.2175/106143015X14212658612993 25842529

[wer70013-bib-0043] Kumari, P. , & Hansdah, P. (2023). Sources and toxicological effects of metal and metalloids on human health through fish consumption in mineral‐rich city, Ranchi, India. Environmental Monitoring and Assessment, 195(9), 1032. 10.1007/s10661-023-11639-2 37561244

[wer70013-bib-0044] Kumwimba, M. N. , Zhu, B. , Wang, T. , Yuan, Z. , & Muyembe, D. K. (2016). Metal distribution and contamination assessment in drainage ditch water in the main rice/vegetable area of Sichuan hilly basin. Bulletin of Environmental Contamination and Toxicology, 96, 248–253. 10.1007/s00128-015-1706-2 26662271

[wer70013-bib-0045] Leventeli, Y. , & Yalcin, F. (2021). Data analysis of heavy metal content in riverwater: Multivariate statistical analysis and inequality expressions. Journal of Inequalities and Applications, 2021, 14. 10.1186/s13660-021-02549-3

[wer70013-bib-0046] Li, S. , Gu, S. , Liu, W. , Han, H. , & Zhang, Q. (2008). Water quality in relation to land use and land cover in the upper Han River basin, China. Catena, 75, 216–222. 10.1016/j.catena.2008.06.005

[wer70013-bib-0047] Li, S. , Xu, Z. , Cheng, X. , & Zhang, Q. (2008). Dissolved trace elements and heavy metals in the Danjiangkou reservoir, China. Environmental Geology, 55, 977–983. 10.1007/s00254-007-1047-5

[wer70013-bib-0048] Lu, G. Y. , & Wong, D. W. (2008). An adaptive inverse‐distance weighting spatial interpolation technique. Computers & Geosciences, 34, 1044–1055. 10.1016/j.cageo.2007.07.010

[wer70013-bib-0049] Ma, Q. L. , Yao, L. A. , Guo, Q. W. , Zhou, G. J. , Liang, R. C. , Fang, Q. L. , Xu, Z. C. , & Zhao, X. M. (2021). Long‐term impact of accidental pollution on the distribution and risks of metals and metalloids in the sediment of the Longjiang River, China. Environmental Science and Pollution Research, 28, 1889–1900. 10.1007/s11356-020-10505-9 32860603

[wer70013-bib-0050] Markad, A. T. , Landge, A. T. , Nayak, B. B. , Inamdar, A. B. , & Mishra, A. K. (2021). A multivariate statistical approach for the evaluation of spatial and temporal dynamics of surface water quality from the small reservoir located in the drought‐prone area of south‐West India: A case study of Tiru reservoir (India). Environmental Science and Pollution Research, 28, 31013–31031. 10.1007/s11356-020-12001-6 33594572

[wer70013-bib-0051] Mohan, S. V. , Nithila, P. , & Reddy, S. J. (1996). Estimation of heavy metals in drinking water and development of heavy metal pollution index. Journal of Environmental Science & Health Part a, 31, 283–289. 10.1080/10934529609376357

[wer70013-bib-0052] Morkoç, E. , Tüfekçi, V. , Tüfekçi, H. , Tolun, L. , Karakoç, F. T. , & Güvensel, T. (2009). Effects of land‐based sources on water quality in the Omerli reservoir (Istanbul, Turkey). Environmental Geology, 57, 1035–1045. 10.1007/s00254-008-1389-7

[wer70013-bib-0053] Muhammad, S. (2023). Evaluation of heavy metals in water and sediments, pollution, and risk indices of Naltar Lakes, Pakistan. Environmental Science and Pollution Research, 30(10), 28217–28226. 10.1007/s11356-022-24160-9 36399291

[wer70013-bib-0054] Muhammad, S. , Zeb, A. , Ullah, R. , Amin, S. , Ahmad, A. , & Tokatli, C. (2024). Spatial distribution of drinking, irrigation water quality, and health risk indices of high‐altitude lakes. Physics and Chemistry of the Earth, Parts a/B/C, 134, 103597. 10.1016/j.pce.2024.103597

[wer70013-bib-0055] Muneer, J. , AlObaid, A. , Ullah, R. , Rehman, K. U. , & Erinle, K. O. (2022). Appraisal of toxic metals in water, bottom sediments and fish of fresh water lake. Journal of King Saud University‐Science, 34(1), 101685. 10.1016/j.jksus.2021.101685

[wer70013-bib-0056] Nguyen, H. , Leermakers, M. , Osán, J. , Török, S. , & Baeyens, W. (2005). Heavy metals in Lake Balaton: Water column, suspended matter, sediment and biota. Science of the Total Environment, 340, 213–230. 10.1016/j.scitotenv.2004.07.032 15752503

[wer70013-bib-0057] Nivesh, S. , Patil, J. P. , Goyal, V. C. , Saran, B. , Singh, A. K. , Raizada, A. , Malik, A. , & Kuriqi, A. (2023). Assessment of future water demand and supply using WEAP model in Dhasan River basin, Madhya Pradesh, India. Environmental Science and Pollution Research, 30(10), 27289–27302. 10.1007/s11356-022-24050-0 36380179

[wer70013-bib-0058] Okoye, E. A. , Bocca, B. , Ruggieri, F. , Ezejiofor, A. N. , Nwaogazie, I. L. , Domingo, J. L. , Rovira, J. , Frazzoli, C. , & Orisakwe, O. E. (2021). Metal pollution of soil, plants, feed and food in the Niger Delta, Nigeria: Health risk assessment through meat and fish consumption. Environmental Research, 198, 111273. 10.1016/j.envres.2021.111273 33989622

[wer70013-bib-0059] Outa, J. O. , Kowenje, C. O. , Avenant‐Oldewage, A. , & Jirsa, F. (2020). Trace elements in crustaceans, mollusks and fish in the kenyan part of Lake Victoria: Bioaccumulation, bioindication and health risk analysis. Archives of Environmental Contamination and Toxicology, 78, 589–603. 10.1007/s00244-020-00715-0 32020255 PMC7136317

[wer70013-bib-0060] Özçelik, Ş. , & Tekin‐Özan, S. (2024). Evaluation of selected heavy metal and selenium pollution in water and sediments of Lake Eğirdir (Isparta/Türkiye) using statistical analysis and pollution indices. Oceanological and Hydrobiological Studies, 53(1), 186–207. 10.26881/oahs-2024.2.09

[wer70013-bib-0061] Pace, C. , Fencl, A. , Baehner, L. , Lukacs, H. , Cushing, L. J. , & Morello‐Frosch, R. (2022). The drinking water tool: A community‐driven data visualization tool for policy implementation. International Journal of Environmental Research and Public Health, 19(3), 1419. 10.3390/ijerph19031419 35162442 PMC8834844

[wer70013-bib-0062] Proshad, R. , Kormoker, T. , Islam, M. S. , & Chandra, K. (2019). Potential health risk of heavy metals via consumption of rice and vegetables grown in the industrial areas of Bangladesh. Human and Ecological Risk Assessment: an International Journal, 26, 921–943. 10.1080/10807039.2018.1546114

[wer70013-bib-0063] Qin, Y. , & Tao, Y. (2022). Pollution status of heavy metals and metalloids in Chinese lakes: Distribution, bioaccumulation and risk assessment. Ecotoxicology and Environmental Safety, 248, 114293. 10.1016/j.ecoenv.2022.114293 36403301

[wer70013-bib-0064] Ravikumar, P. , Aneesul Mehmood, M. , & Somashekar, R. (2013). Water quality index to determine the surface water quality of Sankey tank and Mallathahalli lake, Bangalore urban district, Karnataka, India. Applied Water Science, 3, 247–261. 10.1007/s13201-013-0077-2

[wer70013-bib-0065] Sahoo, M. M. , & Swain, J. B. (2020). Modified heavy metal Poll.Ution index (m‐HPI) for surface water quality in river basins, India. Environmental Science and Pollution Research, 27, 15350–15364. 10.1007/s11356-020-08071-1 32077023

[wer70013-bib-0066] Saleem, M. , Iqbal, J. , & Shah, M. H. (2019). Seasonal variations, risk assessment and multivariate analysis of trace metals in the freshwater reservoirs of Pakistan. Chemosphere, 216, 715–724. 10.1016/j.chemosphere.2018.10.173 30391893

[wer70013-bib-0067] Selker, J. S. , & Assouline, S. (2017). An explicit, parsimonious, and accurate estimate for ponded infiltration into soils using the green and Ampt approach. Water Resources Research, 53, 7481–7487. 10.1002/2017WR021020

[wer70013-bib-0068] Shukla, K. , Kumar, P. , Mann, G. S. , & Khare, M. (2020). Mapping spatial distribution of particulate matter using kriging and inverse distance weighting at supersites of megacity Delhi. Sustainable Cities and Society, 54, 101997. 10.1016/j.scs.2019.101997

[wer70013-bib-0069] Şimşek, A. , & Mutlu, E. (2023). Assessment of the water quality of Bartın Kışla (Kozcağız) dam by using geographical information system (GIS) and water quality indices (WQI). Environmental Science and Pollution Research, 30, 58796–58812. 10.1007/s11356-023-26568-3 36991208

[wer70013-bib-0070] Temel, M. (2006). Benthic alge communities in the coastal part of Ömerli Dam Lake (İstanbul, Turkey). Supplementa Ad Acta Hydrobiologica, 8, 65–77.

[wer70013-bib-0071] Temizer, İ. , Güder, A. , Temel, F. , & Cüce, H. (2018). Antioxidant activities and heavy metal contents of Castanea sativa honey. Global NEST Journal, 20, 541–550. 10.30955/gnj.002628

[wer70013-bib-0072] Tercan, E. , & Dereli, M. A. (2020). Development of a land suitability model for citrus cultivation using GIS and multi‐criteria assessment techniques in Antalya province of Turkey. Ecological Indicators, 117, 106549. 10.1016/j.ecolind.2020.106549

[wer70013-bib-0073] Tezer, A. , Çetin, N. , Onur, A. , Menteşe, E. , Albayrak, İ. , & Cengiz, E. (2015). Final report of the development of Integrated Basin management plan based on ecosystem Services in Ömerli Basin (p. 177). Istanbul Technical University.

[wer70013-bib-0074] Tokatlı, C. , Onur, Ş. G. , Dindar, M. B. , Malafaia, G. , Islam, A. R. M. T. , & Muhammad, S. (2023). Spatial‐temporal variability and probabilistic health risk assessment of fluoride from lentic ecosystem, Türkiye. International Journal of Environmental Analytical Chemistry, 1‐20, 8316–8335. 10.1080/03067319.2023.2198645

[wer70013-bib-0075] Tokatlı, C. , Varol, M. , & Ustaoğlu, F. (2023). Ecological and health risk assessment and quantitative source apportionment of dissolved metals in ponds used for drinking and irrigation purposes. Environmental Science and Pollution Research, 30, 52818–52829. 10.1007/s11356-023-26078-2 36849683

[wer70013-bib-0076] Tokatlı, C. , Varol, M. , Ustaoğlu, F. , & Muhammad, S. (2023). Pollution characteristics, sources and health risks assessment of potentially hazardous elements in sediments of ten ponds in the saros bay region (Türkiye). Chemosphere, 340, 139977. 10.1016/j.chemosphere.2023.139977 37648168

[wer70013-bib-0077] Töre, Y. , Ustaoğlu, F. , Tepe, Y. , & Kalipci, E. (2021). Levels of toxic metals in edible fish species of the Tigris River (Turkey); threat to public health. Ecological Indicators, 123, 107361. 10.1016/j.ecolind.2021.107361

[wer70013-bib-0078] Türker, U. (2002). Ömerli Dam and its effects on the water quality of Ömerli reservoir. Turkish Journal of Engineering and Environmental Sciences, 26(2), 119–127.

[wer70013-bib-0079] Türkmen, A. , Türkmen, M. , Tepe, Y. , & Akyurt, İ. (2005). Heavy metals in three commercially valuable fish species from Iskenderun Bay, northern East Mediterranean Sea, Turkey. Food Chemistry, 91, 167–172. 10.1016/j.foodchem.2004.08.008

[wer70013-bib-0080] Türkmen, M. , Türkmen, A. , Tepe, Y. , Töre, Y. , & Ateş, A. (2009). Determination of metals in fish species from Aegean and Mediterranean seas. Food Chemistry, 113, 233–237. 10.1016/j.foodchem.2008.06.071 26059163

[wer70013-bib-0081] United Nations Development Programme (UNDP) . (2018). Climate resilience and water Management in Istanbul. Retrieved from USEPA website www.undp.org

[wer70013-bib-0082] USEPA . (2008). Environmental Protection Agency, Risk Assessment Guidance for Superfund. In Human health evaluation manual (part a), Washington, DC (Vol. 1).

[wer70013-bib-0083] USEPA , (2018) Integrated risk information system (IRIS) (2018). Retrieved from USEPA website http://www.epa.gov/iris/, Accessed 16th May 2018.

[wer70013-bib-0084] Ustaoğlu, F. , & Aydın, H. (2020). Health risk assessment of dissolved heavy metals in surface water in a subtropical rivers basin system of Giresun (North‐Eastern Turkey). Desalination and Water Treatment, 194, 222–234. 10.5004/dwt.2020.25900

[wer70013-bib-0085] Ustaoğlu, F. , Tepe, Y. , Aydın, H. , & Akbaş, A. (2017). Investigation of water quality and pollution level of lower Melet River, Ordu, Turkey. Alinteri Journal of Agriculture Science, 32, 69–79. 10.28955/alinterizbd.319403

[wer70013-bib-0086] Ustaoğlu, F. , Tepe, Y. , Aydin, H. , & Akbas, A. (2020). Evaluation of surface water quality by multivariate statistical analyses and WQI: Case of comlekci stream,(Giresun‐Turkey). Fresenius Environmental Bulletin, 29, 167–177.

[wer70013-bib-0087] Varol, M. (2013). Dissolved heavy metal concentrations of the Kralkızı, Dicle and batman dam reservoirs in the Tigris River basin, Turkey. Chemosphere, 93, 954–962. 10.1016/j.chemosphere.2013.05.061 23800586

[wer70013-bib-0088] Varol, M. , Ustaoğlu, F. , & Tokatlı, C. (2022). Ecological risks and controlling factors of trace elements in sediments of dam lakes in the Black Sea region (Turkey). Environmental Research, 205, 112478. 10.1016/j.envres.2021.112478 34863685

[wer70013-bib-0089] Varol, S. , Davraz, A. , Şener, Ş. , Şener, E. , Aksever, F. , Kırkan, B. , & Tokgözlü, A. (2021). Assessment of groundwater quality and usability of Salda Lake Basin (Burdur/Turkey) and health risk related to arsenic pollution. Journal of Environmental Health Science and Engineering, 19, 681–706. 10.1007/s40201-021-00638-5 34150267 PMC8172728

[wer70013-bib-0090] Voza, D. , Vukovic, M. , Takic, L. , Nikolic, D. , & Mladenovic‐Ranisavljevic, I. (2015). Application of multivariate statistical techniques in the water quality assessment of Danube river (Vol. 41) (pp. 96–103). Archives of Environmental Protection. 10.1515/aep-2015-0044

[wer70013-bib-0091] Wang, R. , Xia, W. , Eggleton, M. A. , Qu, X. , Liu, H. , Xin, W. , Wu, X. , & Chen, Y. (2022). Spatial and temporal patterns of heavy metals and potential human impacts in Central Yangtze lakes, China. Science of the Total Environment, 820, 153368. 10.1016/j.scitotenv.2022.153368 35077782

[wer70013-bib-0092] Withanachchi, S. S. , Ghambashidze, G. , Kunchulia, I. , Urushadze, T. , & Ploeger, A. (2018). Water quality in surface water: A preliminary assessment of heavy metal contamination of the Mashavera River, Georgia. International Journal of Environmental Research and Public Health, 15, 621. 10.3390/ijerph15040621 29597320 PMC5923663

[wer70013-bib-0093] World Health Organisation (WHO) . (2017). Guidelines for drinking‐water quality: Fourth edition incorporating the first addendum ISBN 978–92–4‐154995‐0 (p. 634). World Health Organization. Licence: CC BY‐NC‐SA 3.0 IGO 28759192

[wer70013-bib-0094] Xiao, J. , Wang, L. , Deng, L. , & Jin, Z. (2019). Characteristics, sources, water quality and health risk assessment of trace elements in river water and well water in the Chinese loess plateau. Science of the Total Environment, 650, 2004–2012. 10.1016/j.scitotenv.2018.09.322 30290343

[wer70013-bib-0095] Xie, Q. , & Ren, B. (2022). Pollution and risk assessment of heavy metals in rivers in the antimony capital of Xikuangshan. Scientific Reports, 12(1), 14393. 10.1038/s41598-022-18584-z 35999241 PMC9399248

[wer70013-bib-0096] Yazman, M. M. , Yüksel, B. , Ustaoğlu, F. , Şen, N. , Tepe, Y. , & Tokatlı, C. (2024). Investigation of groundwater quality in the southern coast of the Black Sea: Application of computational health risk assessment in Giresun, Türkiye. Environmental Science and Pollution Research, 31(39), 52306–52325. 10.1007/s11356-024-34712-w 39143385

[wer70013-bib-0097] Yu, G. , Sun, D. , & Zheng, Y. (2007). Health effects of exposure to natural arsenic in groundwater and coal in China: An overview of occurrence. Environmental Health Perspectives, 115, 636–642. 10.1289/ehp.9268 17450236 PMC1852669

[wer70013-bib-0098] Yüksel, B. , Ustaoğlu, F. , Aydın, H. , Tokatlı, C. , Topaldemir, H. , Islam, M. S. , & Muhammad, S. (2024). Appraisal of metallic accumulation in the surface sediment of a fish breeding dam in Türkiye: A stochastical approach to ecotoxicological risk assessment. Marine Pollution Bulletin, 203, 116488. 10.1016/j.marpolbul.2024.116488 38759467

