# Supplementary material for: Spatial distribution of health risk assessment of a drinking water reservoir exposed to urban agglomeration and industrial lead contamination in Istanbul, Türkiye
Source: Water Environ Res. 2025 Feb 3;97(2):e70013. doi: 10.1002/wer.70013 (PMC11790516; doi:10.1002/wer.70013)
Supplement: Supplementary file 1 — Table S1. Relative weight of each heavy metal. Table S2. Toxicological parameters of the investigated metals used for health risk assessment according to USEPA (2008) (Wang et al., 2017). Table S3. Minimum, maximum and mean concentrations of metal in the Ömerli Dam water. Table S4. Permissible limits for surface waters according to standards of the Turkish Standard Institute surface water regulations, WHO (2017) and USEPA (2008). Table S5. Quality classes according to irrigation water indices (Na%, SAR, and MH). [file WER-97-e70013-s001.docx]

***ANALYSIS OF ECOTOXICOLOGICAL INDICES***

***Water quality assessment by WQI***

WQI is a simple, useful and convenient approach to determine the overall quality of surface/groundwater and its suitability as drinking water. For this reason, it has been broadly used in studies that evaluate the water quality in last decade (Cüce et al., 2022; Varol, 2020; Wang et al., 2017). WQI was calculated using the formula below (1);

$$WQI=\sum\left[ W_{i}\times\left( \frac{C_{i}}{S_{i}} \right) \times100 \right] 1$$

where *Wi = wi / Σwi* is the relative weight (Table S1). The *Wi* value is assigned to 5 as maximum and 1 as minimum, with regards to the relative significant effects of the parameters on human health and their significance in water quality (Ustaoğlu and Aydın, 2020). *C_i_* is the concentrations of the parameters included in the calculation, and *S_i_* is the standard values determined by WHO (2017) in drinking water. In this study, 22 water quality parameters (Na, Mg, K, Ca, Al, Cr, Mn, Fe, Co, Ni, Cu, Zn, As, Cd, Hg, Pb, pH, EC, TDS, BOD_5_, Cl^-^, SO_4_) were included in the WQI calculation. Water quality is evaluated in five different classes according to WQI values. Accordingly: WQI < 50 is excellent; 50 ≤ WQI < 100 is good; 100 ≤ WQI < 200 is poor; 200 ≤ WQI < 300 is very poor; WQI ≥ 300 is undrinkable (Xiao et al., 2019).

***Heavy metal pollution index (HPI)***

The HPI is a useful method for assessing the combined effect of individual heavy metal indicators on the general water quality. For this reason, researchers use the HPI value as a comprehensive instrument to findgeneral water quality derived from heavy metals (Herojeet et al., 2015). HPI was calculated using the following formulas (2-4) (Mohan et al., 1996).

$$HPI=\frac{\sum_{i=1}^{n} \left( Q_{i}W_{i} \right)}{\sum_{i=1}^{n} W_{i}} 2$$

$$Q_{i}=\frac{C_{i}}{S_{i}}x100 3$$

$$W_{i}=\frac{k}{S_{i}} 4$$

*Q_i_* represents the sub-index of each metal, *C_i_* represents the detected concentration value of metals, the standard values of *S_i_* parameters permitted by WHO (2017) as drinking water, *W_i_* represents the unit weight of metals, and k represents a fixed value of "1". If HPI is <100, it indicates a slight level of heavy metal contamination and no adverse related health effects. HPI = 100 indicates threshold risk as well as potential adverse health effects. If HPI is > 100, water is not usable for drinking and also not suitable for consumption (Tokatlı et al., 2023; Xie and Ren, 2022).

***Heavy metal evaluation index (HEI)***

The index of HEI was used as an indicator of heavy metal contamination in water. Hence, it helps the easy interpretation of the water pollution level (Edet and Offiong, 2002). HEI was computed according to the formula below.

$$HEI=\sum_{i=1}^{n} \frac{H_{C}}{H_{MAC}} 5$$

Here, *HC* stands for the value determined for each metal and *H_MAC_* stands for the maximum allowed concentration value (MAC) of each metal (WHO, 2017). If HEI <10, it is interpreted as "low pollution", if 10 <HEI <20 "medium pollution", if HEI> 20 it is interpreted as "high pollution"(Tokatli, 2021).

***Health risks assessments***

Heavy metals from freshwater are taken the human body through ingestion or skin contact. Non-carcinogenic and carcinogenic health effects from oral intake and skin contact may be estimated by experimental models. In the present study, the health risk evaluation method recommended by USEPA (2008) was used and the toxicological parameters of metals are shown in Table S2. The average daily dose (ADD) by direct digestion (ADD ingestion) and skin absorption (ADD dermal) was calculated using the formulas (6 and 7) below (Saleem et al., 2019).

$${ADD}_{ingestion}=\frac{C_{water}\times IR\times{ABS}_{g}\times EF\times ED}{BW\times AT} 6$$

$${ADD}_{dermal}=\frac{C_{water}\times SA\times K_{p}\times ET\times EF\times ED\times CF}{BW\times AT} 7$$

where *ADD_ingestion_* expresses average daily dose by ingestion and *ADD_dermal_* reveals average daily dose by dermal, μg/kg/d; *C_water_* shows level of the heavy metals in freshwater, μg/L; *IR*indicates ingestion rate (L/d), 2 for adult, 0.64 for children in this study; *EF* reveals exposure frequency 365 d/y in this study; *ED* stands for exposure duration (in years), 70 for adults and 6 for children in this study; *K_p_* represents dermal permeability coefficient in water (cm/h); *ET* is the exposure time during bathing and shower, 0.6 h/d in this study; *CF* shows the unit conversion factor, 1 L/1,000 cm^3^; *BW* represents average body weight (kg), 70 for adults and 20 for children in this study; *AT* indicates averaging time (day), 25,550 for adults and 2,190 for children in this study; SA expresses exposed skin area (cm^2^), 18,000 for adults and 6,600 for children in this study; *ABS_g_* which is dimensionless, was the gastrointestinal absorption factor (Xiao et al., 2019).

Hazard quotient (HQ) and hazard index (HI), representing the possible non-carcinogenic effects of heavy metals taken with digestion and skin, were calculated by the following formulas (8 and 9).

$$HQ=\frac{{ADD}_{ingestion}/{ADD}_{dermal}}{{RfD}_{ingestion}/{RfD}_{dermal}} 8$$

$$HI=\sum{HQ}_{s} 9$$

HQ < 1 represents that exposure to any adverse health effects is not likely, while a HI ˃ 1 value indicates that there may be non-carcinogenic effects from heavy metals contact. Carcinogenic risk (CR) describes an individual's risk of cancer due to lifetime contact to potential carcinogens and is calculated by the formula (10) below.

$$CR=ADD\times CSF 10$$

Here; *CSF* is cancer slope factor. Cancer risk was calculated for arsenic only in this study. *CSF* values are 0.0015 and 0.00366 μg/kg/day for digestion and skin, respectively (Gao et al., 2019).

***Water quality evaluation of irrigation water***

Irrigation water quality of the Kızılırmak River was assessed with the SAR, %Na, ,RSC, and MH parameters which calculated as the following formulas (11-13), respectively (Ravikumar et al., 2013).

$$SAR=\frac{{[Na}_{meq}^{+}]}{\sqrt{\frac{{[Ca}_{meq}^{2+}]+{[Mg}_{meq}^{2+}]}{2}}} 11$$

$$Na \%=\frac{\left( {Na}_{meq}^{+}+K_{meq}^{+} \right)\times100}{{Na}_{meq}^{+}+{Ca}_{meq}^{2+}+{Mg}_{meq}^{2+}+K_{meq}^{+}} 12$$

$$MH=\left( \frac{{Mg}_{meq}^{2+}}{{Ca}_{meq}^{2+}+{Mg}_{meq}^{2+}} \right)\times100 13$$

**Table S1.** Relative weight of each heavy metal.

|  | WHO, 2017 | **Assigned Weight (AW)** | **Relative Weight (RW)** |
| --- | --- | --- | --- |
| Na | 200 | 3 | 0.043 |
| Mg | 50 | 2 | 0.029 |
| K | 12 | 2 | 0.029 |
| Ca | 75 | 2 | 0.029 |
| Al | 200 | 4 | 0.058 |
| Cr | 50 | 5 | 0.072 |
| Mn | 400 | 5 | 0.072 |
| Fe | 300 | 1 | 0.014 |
| Co | 50 | 2 | 0.029 |
| Ni | 70 | 5 | 0.072 |
| Cu | 2000 | 2 | 0.029 |
| Zn | 3000 | 1 | 0.014 |
| As | 10 | 5 | 0.072 |
| Cd | 3 | 5 | 0.072 |
| Hg | 6 | 5 | 0.072 |
| Pb | 10 | 5 | 0.072 |
| pH | 7.5 | 3 | 0.043 |
| EC | 1500 | 4 | 0.058 |
| BOD | 5 | 5 | 0.072 |
| Cl | 250 | 3 | 0.043 |
|  |  | 69.0 | 1.0 |

**Table S2.** Toxicological parameters of the investigated metals used for health risk assessment according to USEPA (2008)(Wang et al., 2017)

|  |  | **RfD_ingestion_** | **RfD_dermal_** | **ABS_g_ (%)** |
| --- | --- | --- | --- | --- |
|  | **K_p_** | **(µg/kg/day)** | **(µg/kg/day)** |  |
| Al | 1x10^-3^ | 1000 | 200 | 95 |
| Cr | 1x10^-3^ | 3 | 0.08 | 1.3 |
| Mn | 1x10^-3^ | 24 | 0.96 | 6 |
| Fe | 1x10^-3^ | 700 | 140 | 1.4 |
| Co | 4x10^-4^ | 0.3 | 0.06 | nd |
| Ni | 2x10^-4^ | 20 | 0.8 | 4 |
| Cu | 1x10^-3^ | 40 | 8 | 57 |
| Zn | 6x10^-4^ | 300 | 60 | 20 |
| As | 1x10^-3^ | 0.3 | 0.29 | 95 |
| Cd | 1x10^-3^ | 0.5 | 0.03 | 5 |
| Hg | 1x10^-3^ | 0.3 | 0.02 | 7 |
| Pb | 1x10^-4^ | 1.4 | 0.42 | 11.7 |

**Table S3.** Minimum, maximum and mean concentrations of metal in the Ömerli Dam water.

|  | Wet season | | | Dry season | | | Total | |
| --- | --- | --- | --- | --- | --- | --- | --- | --- |
|  | Mean | Min | Max | Mean | Min | Max | Mean | SD |
| Na (mg/L) | 27.99 | 24.84 | 33.13 | 28.05 | 26.64 | 29.83 | 28.02 | 1.80 |
| Mg (mg/L) | 12.03 | 10.86 | 13.69 | 11.52 | 11.03 | 12.85 | 11.77 | 0.78 |
| K (mg/L) | 6.13 | 5.25 | 7.27 | 5.26 | 4.94 | 5.67 | 5.70 | 0.67 |
| Ca (mg/L) | 65.83 | 59.57 | 74.07 | 61.91 | 57.08 | 76.88 | 63.87 | 5.97 |
| Al (µg/L) | 514.72 | 193.70 | 907.60 | 435.50 | 91.50 | 1312.00 | 475.11 | 355.88 |
| Cr (µg/L) | 4.10 | 1.74 | 7.71 | 3.32 | 1.25 | 8.55 | 3.71 | 2.43 |
| Mn (µg/L) | 96.83 | 58.98 | 183.20 | 74.60 | 13.67 | 340.00 | 85.71 | 90.62 |
| Fe (µg/L) | 518.83 | 249.90 | 1065.00 | 664.73 | 62.14 | 2912.00 | 591.78 | 755.02 |
| Co (µg/L) | 0.84 | 0.04 | 2.61 | 0.61 | 0.08 | 3.24 | 0.72 | 1.03 |
| Ni (µg/L) | 3.25 | 1.30 | 7.77 | 2.41 | 1.11 | 6.24 | 2.83 | 2.00 |
| Cu (µg/L) | 18.16 | 8.13 | 33.59 | 14.82 | 10.54 | 25.58 | 16.49 | 7.08 |
| Zn (µg/L) | 12.22 | 5.13 | 36.85 | 10.64 | 1.40 | 38.21 | 11.43 | 11.68 |
| As (µg/L) | 0.97 | 0.60 | 1.22 | 1.18 | 0.77 | 2.38 | 1.08 | 0.41 |
| Cd (µg/L) | 0.90 | 0.17 | 2.39 | 1.85 | 0.22 | 5.05 | 1.37 | 1.29 |
| Hg (µg/L) | 0.15 | 0.12 | 0.22 | 0.86 | 0.37 | 2.24 | 0.51 | 0.56 |
| Pb (µg/L) | 10.36 | 2.99 | 27.45 | 20.12 | 8.53 | 51.78 | 15.24 | 12.71 |
| WT (°C) | 8.62 | 7.90 | 9.20 | 21.37 | 21.10 | 21.60 | 14.99 | 6.67 |
| pH | 7.81 | 7.24 | 8.21 | 7.98 | 7.32 | 8.17 | 7.89 | 0.34 |
| EC (µS/cm) | 309.33 | 299.00 | 330.00 | 313.83 | 307.00 | 335.00 | 311.58 | 11.67 |
| DO (mg/L) | 9.94 | 9.07 | 10.55 | 8.19 | 7.68 | 8.52 | 9.06 | 1.01 |
| BOD (mg/L) | 3.06 | 2.85 | 3.31 | 7.09 | 2.04 | 13.64 | 5.07 | 3.65 |
| COD (mg/L) | 55.88 | 9.86 | 118.33 | 58.07 | 16.44 | 151.20 | 56.97 | 45.90 |
| Cl^-^ (mg/L) | 61.17 | 51.62 | 75.44 | 67.48 | 60.47 | 77.04 | 64.32 | 8.05 |
| TA (mg/L) | 73.17 | 68.00 | 80.00 | 124.33 | 101.00 | 144.00 | 98.75 | 28.99 |
| Chl_a (μg/L) | 38.57 | 22.30 | 48.60 | 92.00 | 49.60 | 116.10 | 65.28 | 32.70 |
| SRP (mg/L) | 0.04 | 0.03 | 0.05 | 0.05 | 0.03 | 0.13 | 0.05 | 0.03 |
| TN (mg/L) | 36.17 | 30.80 | 48.40 | 19.47 | 14.10 | 28.90 | 27.82 | 10.60 |
| TSS (mg/L) | 5.40 | 3.60 | 7.80 | 10.37 | 8.10 | 12.40 | 7.88 | 3.02 |
| Anionic S. (mg/L) | 0.35 | 0.01 | 0.81 | 0.06 | 0.02 | 0.12 | 0.21 | 0.29 |

**Table S4.** Permissible limits for surface waters according to standards of the Turkish Standard Institute surface water regulations, WHO (2017) and USEPA (2008).

| **PTEs** | **This Study (mean)** | | **Guideline WHO (2017)** | **USEPA (2008)** | **Turkish water quality standards provided drinking water (2019)**  **A1 A2 A3** | | | | **Turkish drinking water regulations**  **(TS 266, 2005)** | |
| --- | --- | --- | --- | --- | --- | --- | --- | --- | --- | --- |
| As (μg/L) | | 1.08 | 10 | 10 | 10 | 40 | 100 | |  | 10 |
| Co (μg/L) | | 0.72 | – | – | 800 | - | 2600 | |  |  |
| Cr (μg/L) | | 3.71 | 50 | 100 | 50 | 500 | 1000 | |  | 50 |
| Cu (μg/L) | | 16.49 | 2000 | 1000 | 2000 | 5000 | 20000 | |  | 2000 |
| Cd (μg/L) | | 1.37 | 3 | 5 | 5 | 15 | 50 | |  | 5 |
| Mn (μg/L) | | 85.71 | 400 | 50 | 50 | 100 | 250 | |  | 50 |
| Ni (μg/L) | | 2.83 | 70 | – | 20 | 30 | 200 | |  | 20 |
| Pb (μg/L) | | 15.24 | 10 | 15 | 10 | 50 | 100 | |  | 10 |
| Fe (μg/L) | | 591.78 | 300 | 300 | 200 | 1000 | 2000 | |  | 200 |
| Al (μg/L) | | 475.11 | 200 | 200 | 200 | 500 | 2000 | |  | 200 |
| Zn (μg/L) | | 11.43 | 5000 | 5000 | 3000 | 6000 | 12000 | |  | 3000 |
| Hg (μg/L) | | 0.51 | 6.0 | 2 | 1 | 2.5 | 5 | |  | 1 |
|  | | |  |  |  |  |  |  |  | |

**Table S5.** Quality classes according to irrigation water indices (Na%, SAR, and MH)

| **MH (meq/L)** | Water quality | **Na%** | Water quality | **SAR (meq/L)** | Water quality |
| --- | --- | --- | --- | --- | --- |
| < 50 | Suitable | < 20 | Excellent | 0–6 | Good |
| > 50 | Unsuitable | 20–40 | Good | 6–9 | Doubtful |
|  |  | 40–60 | Permissible | > 9 | Unsuitable |
|  |  | 60–80 | Doubtful |  |  |
|  |  | > 80 | Unsuitable |  |  |

**References**

Cüce, H., Kalipci, E., Ustaoğlu, F., Dereli, M. A. & Türkmen, A.: 2022, 'Integrated spatial distribution and multivariate statistical analysis for assessment of ecotoxicological and health risks of sediment metal contamination, Ömerli Dam (Istanbul, Turkey)', Water, Air, & Soil Pollution 233, 199.

Edet, A. & Offiong, O. E.: 2002, 'Evaluation of water quality pollution indices for heavy metal contamination monitoring. A study case from Akpabuyo-Odukpani area, Lower Cross River Basin (southeastern Nigeria)', GeoJournal 57, 295-304.

Gao, B., Gao, L., Gao, J., Xu, D., Wang, Q. & Sun, K.: 2019, 'Simultaneous evaluations of occurrence and probabilistic human health risk associated with trace elements in typical drinking water sources from major river basins in China', Science of the Total Environment 666, 139-146.

Herojeet, R., Rishi, M. S. & Kishore, N.: 2015, 'Integrated approach of heavy metal pollution indices and complexity quantification using chemometric models in the Sirsa Basin, Nalagarh valley, Himachal Pradesh, India', Chinese Journal of Geochemistry 34, 620-633.

Mohan, S. V., Nithila, P. & Reddy, S. J.: 1996, 'Estimation of heavy metals in drinking water and development of heavy metal pollution index', Journal of Environmental Science & Health Part A 31, 283-289.

Ravikumar, P., Aneesul Mehmood, M. & Somashekar, R.: 2013, 'Water quality index to determine the surface water quality of Sankey tank and Mallathahalli lake, Bangalore urban district, Karnataka, India', Applied water science 3, 247-261.

Saleem, M., Iqbal, J. & Shah, M. H.: 2019, 'Seasonal variations, risk assessment and multivariate analysis of trace metals in the freshwater reservoirs of Pakistan', Chemosphere 216, 715-724.

Tokatli, C.: 2021, 'Health risk assessment of toxic metals in surface and groundwater resources of a significant agriculture and industry zone in Turkey', Environmental Earth Sciences 80, 156.

Tokatlı, C., Varol, M. & Ustaoğlu, F.: 2023, 'Ecological and health risk assessment and quantitative source apportionment of dissolved metals in ponds used for drinking and irrigation purposes', Environmental Science and Pollution Research 30, 52818-52829.

Ustaoğlu, F. & Aydın, H.: 2020, 'Health risk assessment of dissolved heavy metals in surface water in a subtropical rivers basin system of Giresun (north-eastern Turkey)', Desalination and water treatment 194, 222-234.

Varol, M.: 2020, 'Environmental, ecological and health risks of trace metals in sediments of a large reservoir on the Euphrates River (Turkey)', Environmental Research 187, 109664.

Wang, J., Liu, G., Liu, H. & Lam, P. K.: 2017, 'Multivariate statistical evaluation of dissolved trace elements and a water quality assessment in the middle reaches of Huaihe River, Anhui, China', Science of the total environment 583, 421-431.

Xiao, J., Wang, L., Deng, L. & Jin, Z.: 2019, 'Characteristics, sources, water quality and health risk assessment of trace elements in river water and well water in the Chinese Loess Plateau', Science of the Total Environment 650, 2004-2012.

Xie, Q. & Ren, B.: 2022, 'Pollution and risk assessment of heavy metals in rivers in the antimony capital of Xikuangshan', Scientific reports 12, 14393.
